# Supplementary material for: Targeted KEAP1 disruption enhances antioxidant defense and mesenchymal stromal cell therapy for chronic limb-threatening ischemia
Source: Mol Ther. 2026 Mar 6;34(7):3949–61. doi: 10.1016/j.ymthe.2026.03.005 (PMC13330068; doi:10.1016/j.ymthe.2026.03.005)
Supplement: Supplementary file 1 [file mmc1.pdf]

YMTHE, Volume *34*

Supplemental Information

Targeted *KEAP1* disruption enhances antioxidant defense and mesenchymal stromal cell therapy for chronic limb-threatening ischemia

Eun Ji Shin, Yuri Choi, Eun Je Jeon, Kang-in Lee, Kyu-jun Lee, Young Ju Son, Min Ji Son, Seokjoong Kim, Seung-Woo Cho, and Jae Young Lee

Table S1. sgRNAs used in this study.

sgRNA	Target sequence - PAM (5' to 3')
sgKEAP1_1	TGCAGTCACAGTGCCCTGAG-GGG
sgKEAP1_2	GAAGGTGCGGTTGCCATGCT-GGG
sgKEAP1_3	TGAAGGTGCGGTTGCCATGC-TGG
sgKEAP1_4	GATCATACCAAGCAGGCCTT-TGG
sgKEAP1_5	TTGGCATCATGAACGAGCTG-CGG
sgKEAP1_6	TGAGGCCAGCACCACCTTGT-GGG
sgKEAP1_7	CATGGCCTTGAAGACAGGGC-TGG
sgKEAP1_8	GTGAACATGGCCTTGAAGAC-AGG
sgKEAP1_9	CCATGTTCACCAACGGGCTG-CGG
sgKEAP1_10	ATGGAGGTGGTGTCCATTGA-GGG
sgKEAP1_11	CCAGGTAGCTGAGCGACTGT-CGG
sgKEAP1_12	GAGGCTTACAACCCCAGTGA-CGG
sgKEAP1_13	TCATGGGGTTGTAACAGTCC-AGG
sgKEAP1_14	ACAACCCCATGACCAATCAG-TGG
sgKEAP1_15	CTGCATCCACCACAACAGTG-TGG
sgAAVS1	ATGGAGCCAGAGAGGATCCT-GGG
sgSHS231	GATGTGCTCACTGAGTCTGA-AGG

Table S2. Representative targeted deep-sequencing reads (5 most frequent indel patterns) from sgKEAP1 and Cas9 treated human MSCs. The sgRNA sequence is underlined, the PAM sequence in blue.

Indel	Local Sequence	Frequency (%)
WT	TGCCGTCGGCGGCTCCCACGG**CTGCATCCACCACAACAGTG**TGGAGAGGTGAGTGGCAGGGCCTGGGGGT	
-1	TGCCGTCGGCGGCTCCCACGGCTGCATCCACCACA--AGTGTGGAGAGGTGAGTGGCAGGGCCTGGGGGT	57.51
-2	TGCCGTCGGCGGCTCCCACGGCTGCATCCACCACAACA**A**GTGTGGAGAGGTGAGTGGCAGGGCCTGGGGGT	13.27
+1	TGCCGTCGGCGGCTCCCACGGCTGCATCCACC---ACAGTGTGGAGAGGTGAGTGGCAGGGCCTGGGGGT	3.90
-3	TGCCGTCGGCGGCTCCCACGGCTGCATCCAC-----CAGTGTGGAGAGGTGAGTGGCAGGGCCTGGGGGT	3.08
-4	TGCCGTCGGCGGCTCCCACGGCTGCATCCACCACAAC-GTGTGGAGAGGTGAGTGGCAGGGCCTGGGGGT	0.75

Table S3. Potential off-target sites of the sgKEAP1 in the human genome (GRCh38.hg38) revealed by *in silico* analysis.

	Location	Position	Target sequence (5' to 3')	Gene
On-target	Chr19	10491580	CTGCATCCACCACAACAGTGTGG	*KEAP1*
Off1	Chr3	70902810	CTGCATCCACCACcACAGTGAAG	Intergenic
Off2	Chr7	33225786	CTctATCCACCACAACAGTGCAG	BBS9 (Intron)

Table S4. Potential off-target sites of the sgKEAP1 in the human genome (GRCh38.hg38) revealed by *Digenome-seq* analysis.

	Location	Position	Target sequence (5' to 3')	Gene
On-target	chr19	10491580	CTGCATCCACCACAACAGTGTGG	*KEAP1*
Off3	Chr8	135488419	CCCTACTGTGTGGTGGATACAA	KHDRBS3 (Intron)
Off4	Chr7	33225793	CTGCACTGTTGTGGTGGATAGAG	BBS9 (Intron)
Off5	Chr5	18717963	CCCCACTGTTGTTATGATGCAG	Intergenic
Off6	Chr5	128243049	CCCCACTGTTTGGTGGGTGCAG	Intergenic
Off7	Chr4	72924759	CCCCAATCCCCACAACAGTGTGG	Intergenic
Off8	Chr3	117724462	CTTAATCCCCACAACAATGAGG	ENSG00000242816 (Intron)
Off9	Chr14	61334146	CCACCTCAGTGTGTGGTGGAGGCAG	PRKCH (Intron)

Table S5. Sequence of primers used for RTq-PCR.

Gene	Forward (5′-3′)	Reverse (5′-3′)
human *KEAP1*	ATTGGCTGTGTGGAGTTGC	CAGGTTGAAGAACTCCTCTTGC
human *GAPDH*	AGCCACATCGCTCAGACACC	GTACTCAGCGGCCAGCATCG
human *PRDX1*	CTGCCAAGTGATTGGTGCTTCTG	AATGGTGCGCTTCGGGTCTGAT
human *GCLM*	TCTTGCCTCCTGCTGTGTGATG	TTGGAAACTTGCTTCAGAAAGCAG
human *PTGS2*	CGGTGAAACTCTGGCTAGACAG	GCAAACCGTAGATGCTCAGGGA
human *AKR1C3*	CCGAAGCAAGATTGCAGATGGC	GTGAGTTTTCCAAGGCTGGTCG
human *PPIF*	CGACTTCACCAACCACAATGGC	GGTGTTAGGACCAGCATTAGCC
human *BNIP3*	TCAGCATGAGGAACACGAGCGT	GAGGTTGTCAGACGCCTTCCAA
human *FTH1*	TGAAGCTGCAGAACCAACGAGG	GCACACTCCATTGCATTCAGCC
human *TXNRD1*	GTTACTTGGGCATCCCTGGTGA	CGCACTCCAAAGCGACATAGGA
human *NQO1*	CCTGCCATTCTGAAAGGCTGGT	GTGGTGATGGAAAGCACTGCCT
human *SRXN1*	GCAGAGCCTCGTGGACACGAT	ATGGTCTCTCGCTGCAGTTGCT
human *PRNP*	TTCGGCAGTGACTATGAGGACC	TTGTGGTGACCGTGTGCTGCTT
human *SQSTM1*	TGTGTAGCGTCTGCGAGGGAAA	AGTGTCCGTGTTTCACCTTCCG
human *GSR*	TATGTGAGCCGCCTGAATGCCA	CACTGACCTCTATTGTGGGCTTG
human *TXN*	GTAGTTGACTTCTCAGCCACGTG	CTGACAGTCATCCACATCTACTTC
mouse *Gapdh*	TCACCACCATGGAGAAGGC	GCTAAGCAGTTGGTGGTGCA
mouse *Il10*	CGGGAAGACAATAACTGCACCC	CGGTTAGCAGTATGTTGTCCAGC
mouse *Tgfb1*	TGATACGCCTGAGTGGCTGTCT	CACAAGAGCAGTGAGCGCTGAA
mouse *Mrc1*	GTTCACCTGGAGTGATGGTTCTC	AGGACATGCCAGGGTCACCTTT
mouse *Hgf*	GTCCTGAAGGCTCAGACTTGGT	CCAGCCGTAAATACTGCAAGTGG

Table S6. Donor information for human BM-MSCs used for current study

Vendor	CAT#	LOT#	Stock passage	Age(Y)	Sex	Race	>=Positive 90%	5%<=positive
Lonza	PT-2501	19TL155677	P2	31	M	Black	CD29 44 73 90 105 166	CD31 45 DR 19
Lonza	PT-2501	20TL153402	P2	35	M	Caucasian	CD29 44 73 90 105 166	CD14 31 45 DR 19
Lonza	PT-2501	21TL333343	P2	25	M	Black	CD29 73 90 105 166	CD14 31 45 DR 19

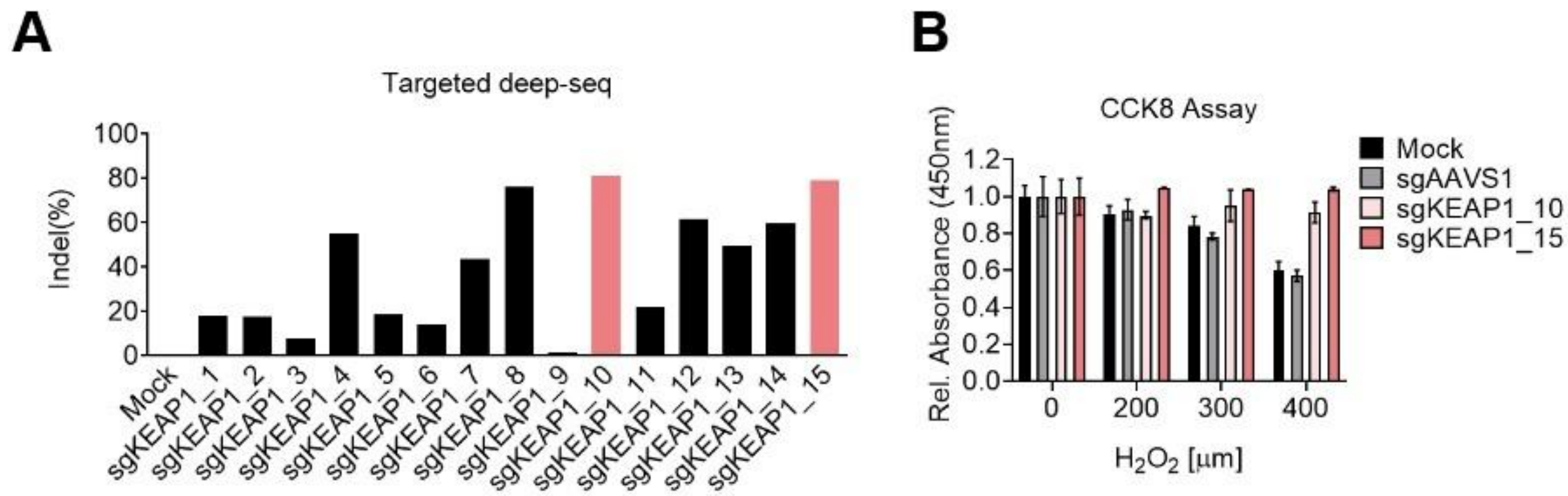

Figure S1. Screening and identification of optimal sgRNA for *KEAP1* gene disruption.

(A) Targeted deep sequencing results showing indel frequencies induced by 15 sgRNAs designed against *KEAP1* in human bone marrow derived MSCs. sgKEAP1_10 and sgKEAP1_15 exhibited the highest editing efficiencies (highlighted in pink) and were selected for downstream validation. **(B)** Cell viability of sgKEAP1_10– and sgKEAP1_15–edited cells evaluated by CCK-8 assay following exposure to increasing concentrations of hydrogen peroxide (*n* = 2 Mock-transfected and sgAAVS1-edited cells served as controls).

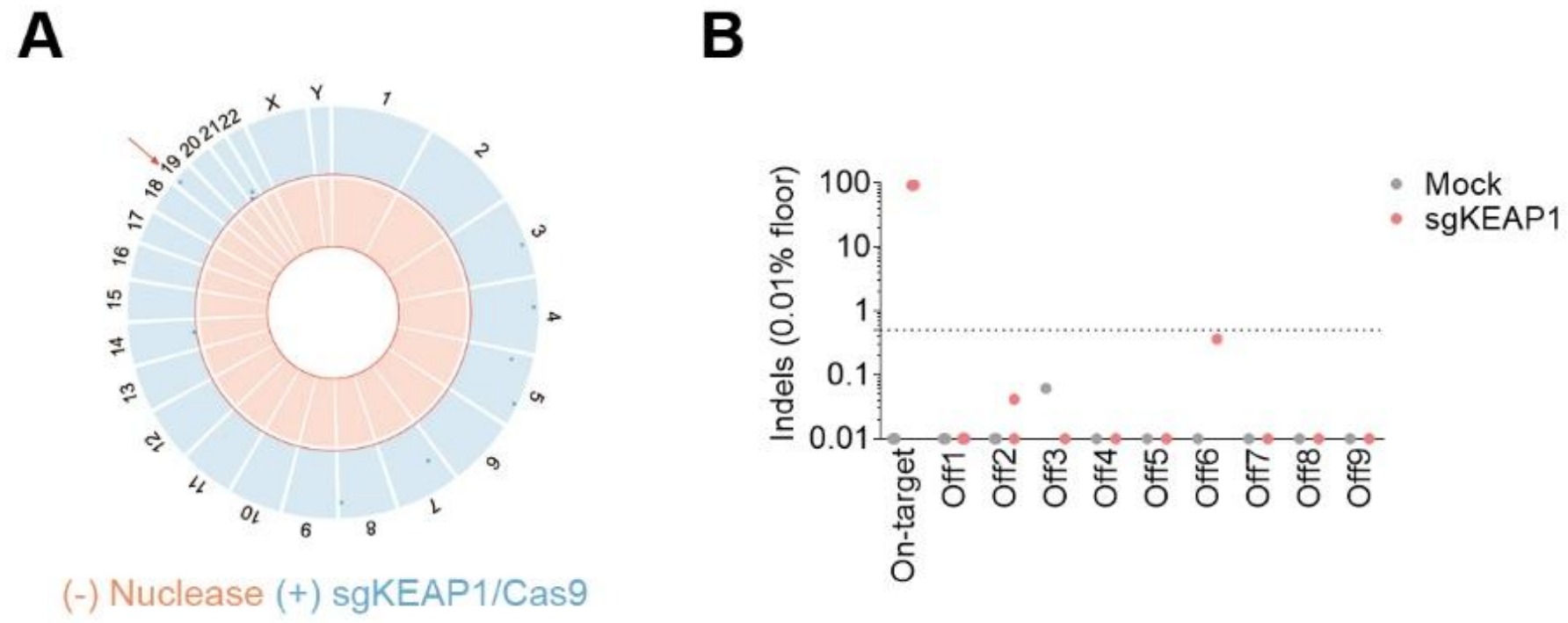

Figure S2. Specificity assessment of *KEAP1* gene editing by sgKEAP1/Cas9.
The overall workflow for off-target evaluation involved genome-wide identification of potential Cas9 cleavage sites by Digenome-seq, followed by targeted deep sequencing to quantitatively validate editing specificity at the on-target and predicted off-target loci. **(A)** Genome-wide off-target profiling by Digenome-seq analysis showing cleavage patterns in the presence (+, blue) or absence (–, orange) of sgKEAP1/Cas9 nuclease. On-target locus on chromosome 19 is indicated in red arrow. **(B)** Targeted deep sequencing of the *KEAP1* on-target site and 9 candidate off-target sites in human BM-MSCs transfected with sgKEAP1 or mock control. Off-target loci were selected based on combined Digenome-seq and *in silico* prediction (≤2 bp mismatch to the sgKEAP1 protospacer). Indel frequencies at all putative off-target sites remained below background threshold (<0.5%), confirming the high specificity of sgKEAP1/Cas9 editing in MSCs.

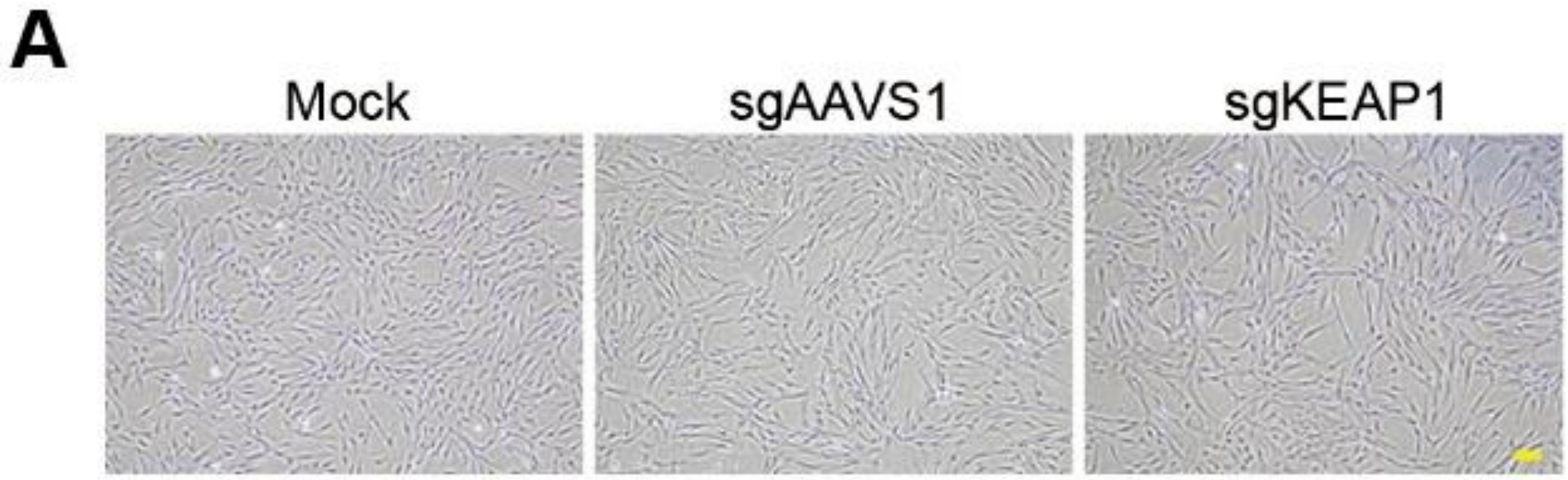

Figure S3. Morphological assessment of MSCs following KEAP1 gene editing.
(A) Representative phase-contrast micrographs of human bone marrow–derived MSCs at day 7 post gene editing. Cells edited with non-targeting control sgAAVS1 or *KEAP1*-specific sgRNA (sgKEAP1) displayed typical fibroblast-like morphology comparable to mock-transfected controls, indicating that *KEAP1* knockout did not alter MSC morphology or gross cellular phenotype. Scale bar, 100 µm.

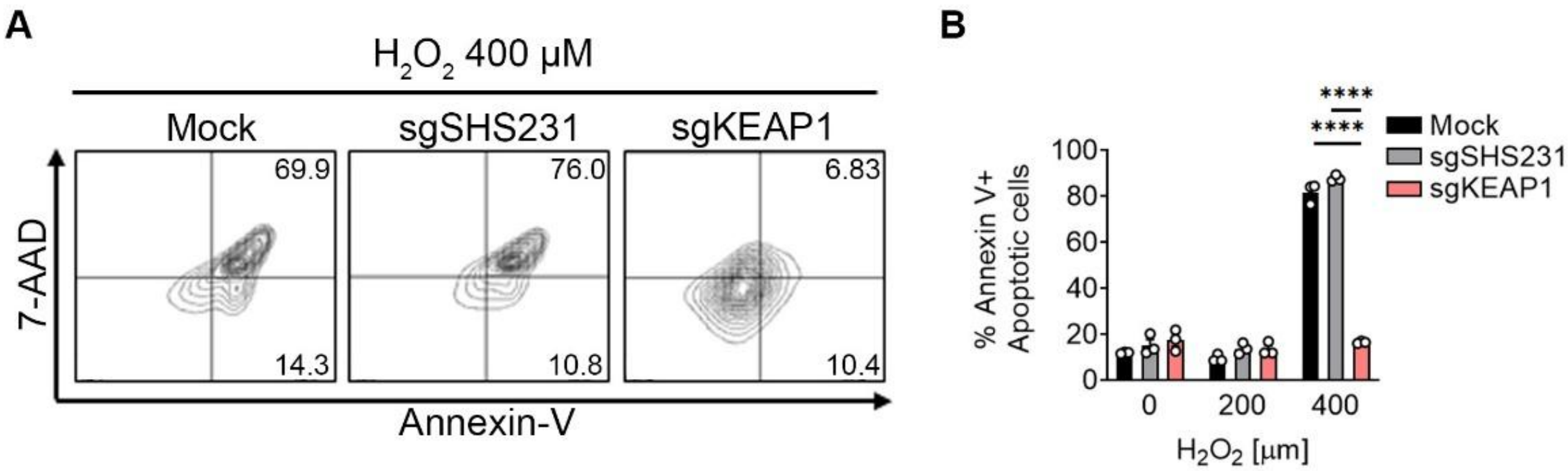

Figure S4. KEAP1 disruption reduces oxidative stress-induced apoptosis in MSCs.
(A) Representative flow cytometric plots of Annexin-V and 7-AAD staining in Mock, sgSHS231, and sgKEAP1 MSCs after 24-hour exposure to 400 μM H_2O_2. (B) Quantification of Annexin-V^+ apoptotic cells across increasing H_2O_2 concentrations $n = 3$, ****$P < 0.0001$ by two-way ANOVA with Tukey's post hoc test).

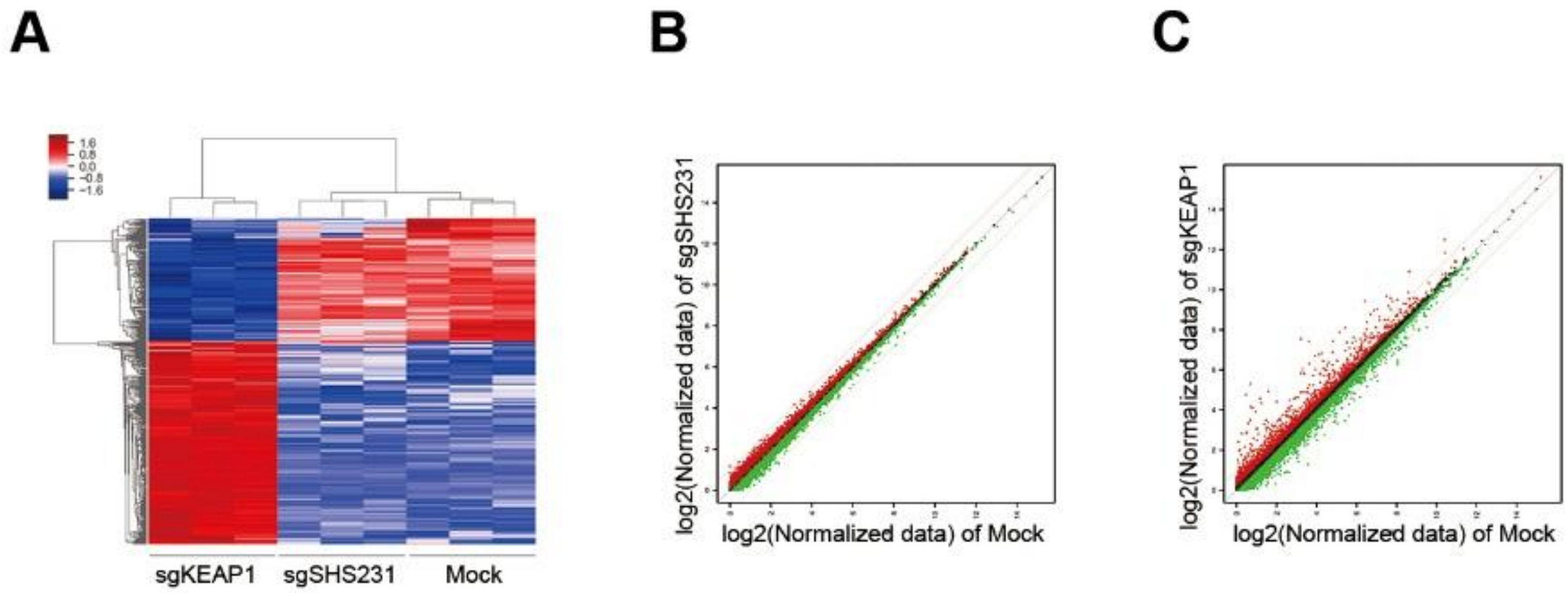

Figure S5. Transcriptomic profiling of KEAP1-edited MSCs by RNA sequencing.
(A) Heatmap displaying hierarchical clustering of differentially expressed genes among *KEAP1*-knockout (sgKEAP1), control (sgSHS231), and mock-transfected MSCs at day 7 post-editing. Each row represents an individual gene, and color scale denotes z-score–normalized expression values (red, upregulated; blue, downregulated). *KEAP1* knockout resulted in a distinct transcriptomic signature characterized by widespread upregulation of antioxidant and cytoprotective genes relative to controls. **(B–C)** Scatter (MA) plots comparing global gene expression profiles of sgSHS231 **(B)** and sgKEAP1 **(C)** versus mock controls. Each dot represents one gene; red and green indicate significantly upregulated and downregulated genes, respectively (fold change >2, adjusted $P<0.05$). *KEAP1* editing induced a broader and more pronounced transcriptomic shift than the non-targeting control, consistent with NRF2 pathway activation and metabolic reprogramming in MSCs.

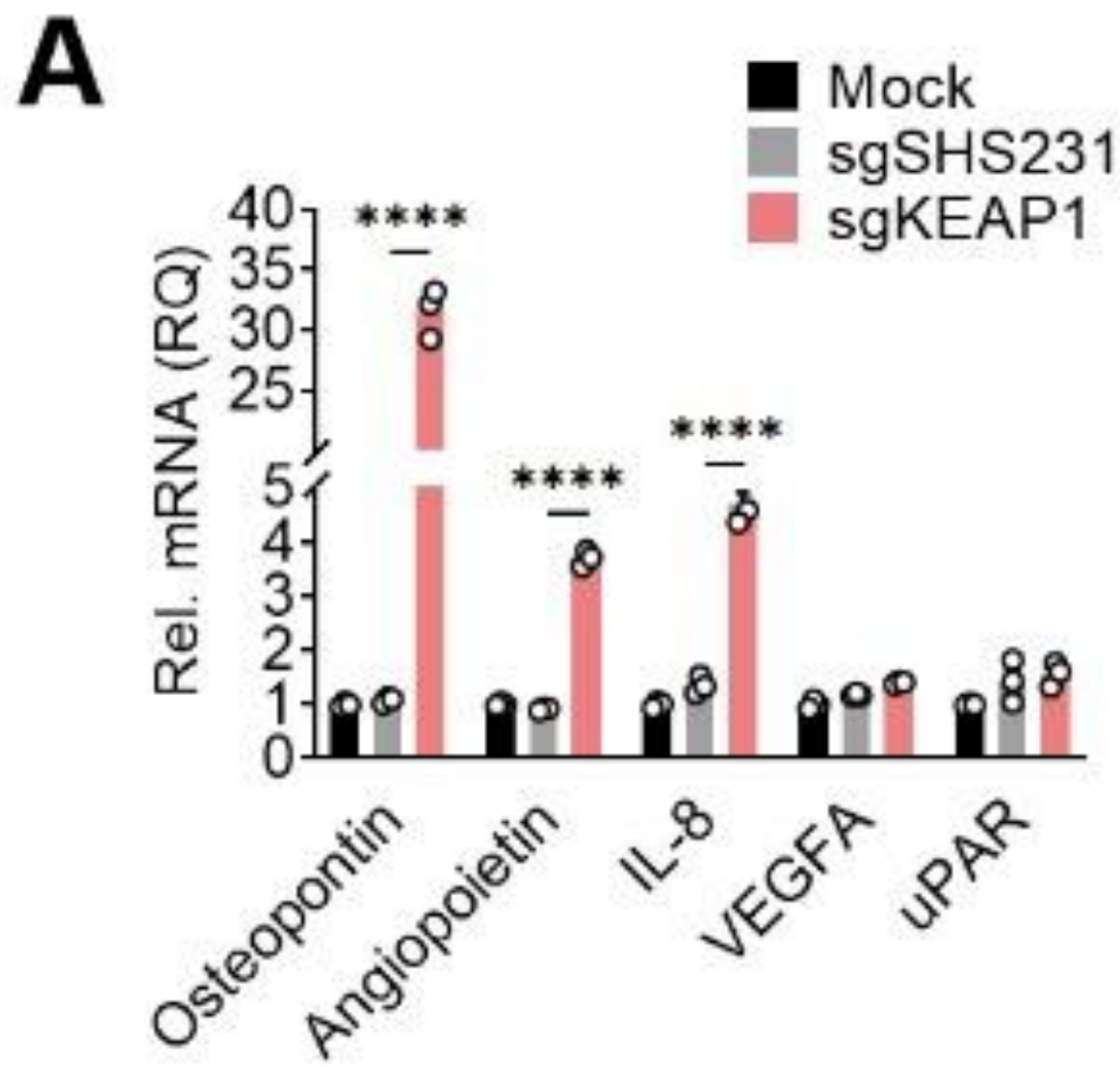

Figure S6. Confirmation of cytokine gene upregulation in sgKEAP1 MSCs.
(A) RT-qPCR analysis of mRNA expression levels corresponding to cytokines identified in Fig. 4 (n = 3 biological replicates; one-way ANOVA with Tukey's post hoc test; ***P < 0.0001).

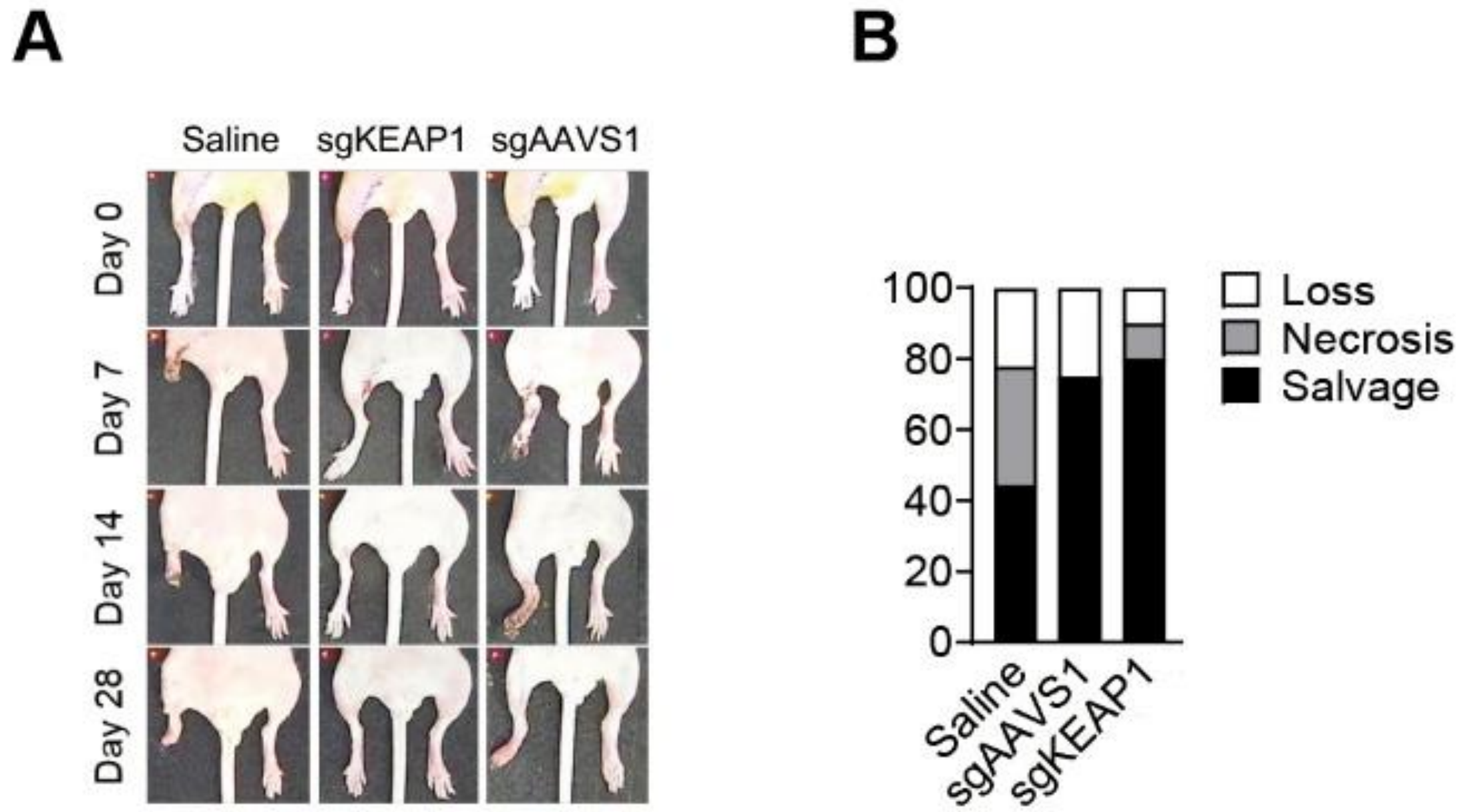

Figure S7. KEAP1-edited MSCs attenuate ischemic tissue necrosis *in vivo*.
(A) Representative macroscopic images of hindlimbs from each treatment group at indicated time points (days 0–28). **(B)** Quantification of limb salvage outcomes based on necrosis scoring at day 28. sgKEAP1-treated limbs exhibited a higher rate of tissue salvage and reduced necrosis compared with sgAAVS1 and saline controls (*n* = 8-10 mice per group).
