# Supplementary material for: Targeted KEAP1 disruption enhances antioxidant defense and mesenchymal stromal cell therapy for chronic limb-threatening ischemia
Source: Mol Ther. 2026 Mar 6;34(7):3949–61. doi: 10.1016/j.ymthe.2026.03.005 (PMC13330068; doi:10.1016/j.ymthe.2026.03.005)
Supplement: Supplementary file 2 [file mmc2.pdf]

Molecular Therapy
Original Article

Targeted *KEAP1* disruption enhances antioxidant defense and mesenchymal stromal cell therapy for chronic limb-threatening ischemia

Eun Ji Shin,[1] Yuri Choi,[1] Eun Je Jeon,[2] Kang-in Lee,[1] Kyu-jun Lee,[1] Young Ju Son,[1] Min Ji Son,[1] Seokjoong Kim,[1] Seung-Woo Cho,[2,3,4] and Jae Young Lee[1,5,6]

[1]ToolGen, Inc., Seoul, Republic of Korea; [2]Cellartgen, Inc., Seoul 03722, Republic of Korea; [3]Department of Biotechnology, Yonsei University, Seoul 03722, Republic of Korea; [4]Center for Nanomedicine, Institute for Basic Science (IBS), Seoul 03722, Republic of Korea; [5]Department of Health Science and Technology, Samsung Advanced Institute of Health Sciences and Technology (SAIHST), Sungkyunkwan University, Seoul 06351, Republic of Korea; [6]Cell and Gene Therapy Institute (CGTI), Research Institute for Future Medicine, Samsung Medical Center, Seoul 06351, Republic of Korea

Chronic limb-threatening ischemia (CLTI) is a severe vascular disorder characterized by tissue hypoxia and oxidative stress that limit the efficacy of regenerative therapies. Mesenchymal stem/stromal cells (MSCs) hold promise for CLTI treatment through paracrine angiogenic and immunomodulatory signaling, yet their survival and function are compromised in the reactive oxygen species-rich ischemic microenvironment. Here, we utilized CRISPR-Cas9 to generate a targeted knockout of *Kelch-like ECH-associated protein 1* (*KEAP1*), the negative regulator of the antioxidant transcription factor NRF2, in human bone marrow-derived MSCs. *KEAP1* editing activated the NRF2 pathway, reduced intracellular oxidative stress, and reprogrammed redox and paracrine gene networks. Edited MSCs exhibited enhanced viability, sustained secretion of proangiogenic cytokines, and improved tissue perfusion and arteriogenesis in a murine model of CLTI. These findings establish *KEAP1* gene editing as a permanent, integration-free strategy to augment MSC resistance and therapeutic efficacy in oxidative ischemic environments.

INTRODUCTION

Chronic limb-threatening ischemia (CLTI), the end stage of peripheral arterial disease, remains a major cause of morbidity and amputation worldwide despite advances in surgical and endovascular therapies.[1–5] The ischemic microenvironment of CLTI is characterized by hypoxia, inflammation, and excessive reactive oxygen species (ROS), which cause endothelial injury, impair neovascularization, and lead to irreversible tissue loss.[6–10] Both clinical and experimental studies have demonstrated that ischemic limbs exhibit sustained oxidative stress that limits endogenous repair mechanisms and compromises cell-based therapies.[11,12] Hence, enhancing antioxidant defenses may represent a rational strategy to improve tissue recovery and therapeutic outcomes in CLTI.

Mesenchymal stem/stromal cells (MSCs) have emerged as a promising cell source for treating ischemic diseases due to their pro-angiogenic and anti-inflammatory effects through paracrine activity.[13,14] MSCs can secrete pro-angiogenic factors such as vascular endothelial growth factor (VEGF), as well as angiopoietin-1 (ANG-1) to promote the vascularization process and release beneficial cytokines to enhance tissue repair.[14] However, the oxidative milieu in ischemic tissue can rapidly deplete the antioxidant capacity of transplanted MSCs, leading to poor survival and loss of therapeutic potency.[15,16] Therefore, augmenting the redox resilience of MSCs is critical for achieving durable benefit in CLTI.

Several approaches have been explored to enhance MSC antioxidant capacity.[17–23] Overexpression of *NRF2*, the master regulator of the antioxidant response, protects MSCs against oxidative injury, promotes angiogenic factor secretion, and enhances recovery in ischemic models.[17–20] Likewise, silencing of *Kelch-like ECH-associated protein 1* (*KEAP1*), the cytoplasmic repressor of NRF2 upregulates antioxidant genes.[21–23] However, most current methods such as hypoxic or cytokine "priming," transient small interfering RNA (siRNA), or viral overexpression can pose limitations. Culture-based priming can transiently activate NRF2 signaling but can lose efficacy upon transplantation.[24] RNA interference (RNAi) such as siRNA-based silencing is short-lived and loses efficacy upon MSC proliferation, while the lentiviral or retroviral mediated small hairpin RNA approach carries risks of random integration, variable transgene expression, and potential insertional mutagenesis.[25] Thus, a permanent and precise genome-editing approach that enhances endogenous antioxidant signaling without exogenous DNA integration may overcome these challenges.

Received 20 November 2025; accepted 3 March 2026;
https://doi.org/10.1016/j.ymthe.2026.03.005.

Correspondence: Seung-Woo Cho, Department of Biotechnology, Yonsei University, Seoul 03722, Republic of Korea.
E-mail: seungwoocho@yonsei.ac.kr
Correspondence: Jae Young Lee, Department of Health Science and Technology, Samsung Advanced Institute of Health Sciences and Technology (SAIHST), Sungkyunkwan University, Seoul 06351, Republic of Korea.
E-mail: jyjlee0930@gmail.com

© 2026 The Author(s).
Published by Elsevier Inc. on behalf of The American Society of Gene and Cell Therapy.
This is an open access article under the CC BY license (http://creativecommons.org/licenses/by/4.0/).

CRISPR-Cas9-mediated gene editing enables efficient and specific modification of endogenous loci, providing a stable alternative to transient manipulation. Deletion of *KEAP1* by CRISPR-Cas9 has been shown to stabilize NRF2 and upregulate antioxidant enzymes in human T cells and rat MSCs.[26] However, its potential to enhance human MSC-based therapy for ischemic disease has not been comprehensively investigated, particularly in the context of clinically relevant human MSC-based therapy.

Here, we hypothesized that targeted knockout of *KEAP1* in human MSCs would activate the NRF2-dependent antioxidant network, thereby improving cell survival, paracrine function, and therapeutic efficacy in oxidative ischemic environments. To test this, we established efficient and specific *KEAP1* editing using CRISPR-Cas9 and assessed oxidative resilience *in vitro*. We then investigated transcriptome and secretome remodeling, focusing on antioxidant and angiogenic pathways. Finally, using a mouse model of hindlimb ischemia, we evaluated whether *KEAP1*-edited MSCs exhibit enhanced survival and vascular regeneration compared to controls. Collectively, this work identifies a precise and durable genome-editing strategy to augment MSC antioxidant capacity and establishes *KEAP1* disruption as a rational approach to improve cell therapy efficacy in ROS-rich ischemic disease.

RESULTS

Efficient and specific KEAP1 gene editing in human MSCs

To generate *KEAP1*-deficient MSCs, we designed and prepared 20 single-guide RNAs (sgRNAs) targeting human *KEAP1* exons (Table S1) by *in vitro* transcription and screened for editing efficiency in MSCs by transfecting sgRNA-Cas9 ribonucleoprotein (RNP) complexes using a targeted deep sequencing (Figures S1A and S1B). Among these, sgKEAP1_10 and sgKEAP1_15 exhibited the highest editing efficiency. To select the lead sgRNA, we performed cell viability assays under oxidative stress conditions and found that sgKEAP1_15 showed better cell survival than sgKEAP1_10-edited MSCs and was selected for subsequent experiments (Figure S1B; sgKEAP1_15 hereafter referred to as sgKEAP1). SHS231 and AAVS1 safe harbor sites targeting sgRNAs (sgSHS231 and sgAAVS1) were utilized as controls.[27,28]

We then synthesized chemically modified sgRNAs and transfected with Cas9 as RNP complexes in MSCs, which resulted in efficient insertions or deletions (indels) at the expected Cas9 cleavage site (Figures 1A and 1B; Table S2). To determine whether sgKEAP1-edited MSC led to a reduction in *KEAP1* gene expression, we performed quantitative reverse transcription PCR (RT-qPCR) analysis and found a significant reduction in *KEAP1* transcript compared with controls (Figure 1C). We also performed western blot analysis and demonstrated a trend toward reduction in KEAP1 protein and a concomitant increase in the active form of NRF2; phosphorylated NRF2, suggesting NRF2 pathway activation (Figure 1D). To analyze the specificity of the sgKEAP1, we performed *in silico*-based on-target analysis, which showed two potential off-target sites containing up to 2 bp mismatches (NRG as protospacer-adjacent motif [PAM]; R = A or G) in the human reference genome (Table S3). As *in silico*-based on-target analysis can be biased,[29] we also performed Digenome-seq, an unbiased whole-genome sequencing-based on-target analysis[30] and found seven potential off-target sites (Figure S2; Table S4). However, re-analysis by targeted deep sequencing in sgKEAP1 MSCs showed verified on-target editing at *KEAP1* and absence of detectable indels in 10 potential off-target loci identified *in silico* and Digenome-seq (Figure S2B), confirming high specificity.

Importantly, *KEAP1* editing did not alter MSC morphology, viability, population doubling time (PDT), or expression of characteristic MSC surface markers CD73, CD90, and CD105 upon long-term culture (Figures 1E–1H and S3). We also tracked for long-term stability of *KEAP1* gene editing and found no change in indels induced at *KEAP1* loci (Figure 1I), indicating that targeted *KEAP1* editing in MSCs did not impair MSC proliferation or characteristics.

KEAP1 editing enhances MSC resistance to oxidative tress

To evaluate whether *KEAP1* disruption conferred protection from high oxidative stress, edited and control MSCs were challenged with hydrogen peroxide (H_2O_2, 300–500 μM, 24 h). Cell viability measured by the Cell Counting Kit-8 (CCK-8) assay revealed significantly greater survival in sgKEAP1 MSCs up to 450 μM H_2O_2 than in mock or sgSHS2331 controls (Figure 2A). Consistent with these findings, annexin V/7-amino-actinomycin D (7-AAD) flow cytometry demonstrated a markedly lower proportion of apoptotic and necrotic cells in sgKEAP1 MSCs compared with controls under oxidative stress (Figures S4A and S4B). Intracellular ROS levels, measured using CellROX fluorescent dye, were markedly lower in sgKEAP1 MSCs under oxidative challenge (Figure 2B). Quantitative image analysis showed reduced mean fluorescence intensity and a lower percentage of ROS^+ cells compared with controls (Figures 2C and 2D), indicating that *KEAP1* deletion alleviates oxidative stress. We then performed RNA sequencing (RNA-seq) of edited and control MSCs to compare the RNA expression between sgKEAP1 and sgSHS231 MSCs (Figures S5A–S5C). Gene Ontology (GO) enrichment analysis highlighted "oxidation-reduction process" and "response to oxidative stress" among the most upregulated biological processes (Figure 2E). Especially in genes in the GO term "response to oxidative stress," robust upregulation of NRF2-dependent antioxidant genes was found in sgKEAP1 compared with sgSHS231 MSCs (Figure 2F). RT-qPCR analysis confirmed significant induction of antioxidant target genes identified in Figure 2F (Figure 2G) in sgKEAP1 when compared with sgSHS231 control in agreement with prior studies showing that KEAP1-NRF2 signaling governs redox equilibrium and cytoprotection in MSCs.[31]

KEAP1 editing remodels the MSC paracrine secretome

Given that MSC efficacy is largely mediated by secreted factors,[32,33] we next profiled cytokines released by sgKEAP1 MSCs 7 days post-editing. The cytokine array showed a trend toward increased secretion of proangiogenic, and immunomodulatory molecules that were found to play a crucial role in limb ischemia, including VEGFA,[34,35] osteopontin,[36,37] ANG-1,[38,39] interleukin-8 (IL-8),[40] and urokinase-type

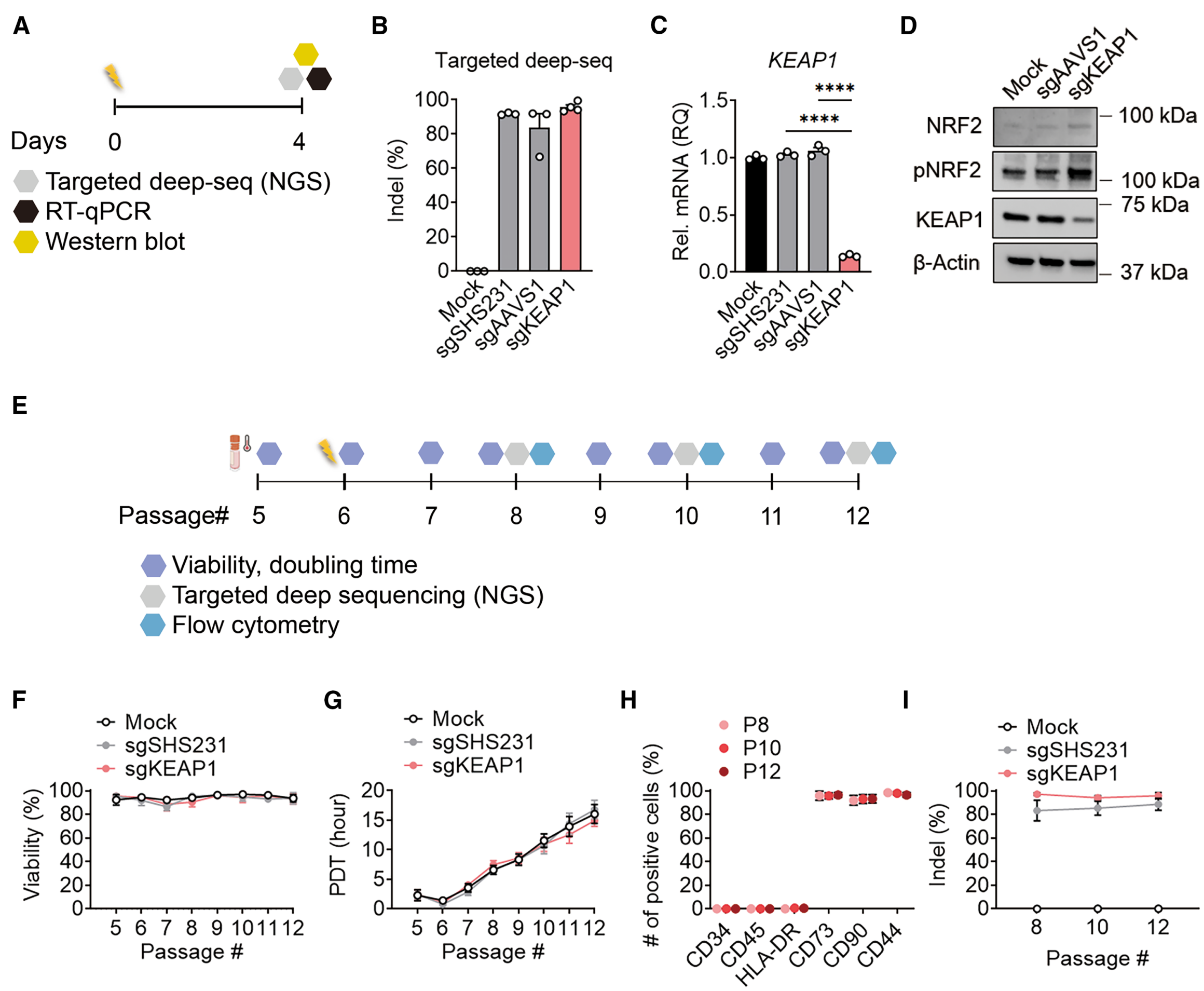

Figure 1. Efficient CRISPR-Cas9-mediated disruption of KEAP1 in human mesenchymal stem cells without alteration of cellular phenotype or proliferation

(A) Schematic illustration of the experimental workflow for (B)–(D). Bone marrow-mesenchymal stem cells (BM-MSCs) were electroporated with CRISPR-Cas9 ribonucleoprotein (RNP) complexes targeting *KEAP1* (sgKEAP1) or non-targeting control (sgSHS231 or sgAAVS1). Cells were harvested at the indicated time points for targeted deep sequencing (next-generation sequencing) and RT-qPCR. (B) Targeted deep sequencing results showing insertion or deletion (indel) frequencies at the *KEAP1* locus 4 days post-editing. sgSHS231, sgAAVS1, and mock-transfected cells served as controls (n = 3–4 biological replicates). (C) RT-qPCR analysis of *KEAP1* mRNA expression normalized to *GAPDH* (one-way ANOVA with Tukey's post hoc test (****$p < 0.0001$, n = 3 biological replicates). (D) Western blot analysis of NRF2, phosphor-NRF2 (pNRF2), KEAP1, and β-actin in mock, sgSHS231, or sgKEAP1 (n = 1). (E) Schematic illustration of the experimental workflow for (F)–(I). (F) Cell viability measured by trypan blue exclusion across serial passages. (G) Population doubling time of edited and control MSCs across passages. (I) Flow cytometric analysis of MSC markers (CD34^{-}, CD45^{-}, HLA-DR^{-}, CD73^{+}, CD90^{+}, and CD44^{+}) at passages 8, 10, and 12. (I) Indel frequencies at the *KEAP1* locus maintained across passages (n = 3 biological replicates).

plasminogen activator receptor[41] relative to controls (Figures 3A and 3B). Enzyme-linked immunosorbent assay (ELISA) confirmed significantly increased VEGFA, osteopontin, and ANGPT1 levels in the conditioned medium of sgKEAP1 MSCs (Figures 3C–3E). Corresponding mRNA levels were upregulated as verified by RT-qPCR (Figure S6), suggesting transcriptional activation of angiogenic and tissue-repair genes downstream of NRF2 activation.

KEAP1-edited MSCs exhibit improved survival and immunomodulatory activity in ischemic tissue

Having achieved increased survival under oxidative stress environment and enhanced paracrine effect for angiogenesis, we then examined sgKEAP1 MSC persistence in the ischemic limb microenvironment. For this, DiR-labeled edited MSCs were injected intramuscularly into ischemic limbs of mice subjected to femoral artery ligation (Figure 4A). *In vivo* fluorescence imaging on day 3 post-injection showed significantly stronger DiR signal intensity in limbs receiving sgKEAP1 MSCs compared with sgAAVS1 or mock controls (Figures 4B and 4C). Quantitative human Alu-specific PCR from mouse ischemic tissue corroborated increased retention of sgKEAP1 MSCs (Figure 4D), consistent with enhanced oxidative stress resistance. Given the increased survival of sgKEAP1 MSCs, we then analyzed whether there is an increased signal of immune modulation. Analysis of host immune response revealed the upregulation of M2 macrophage markers (*Il10*, *Tgfb*, and *Mrc1*) and increased tissue repair gene

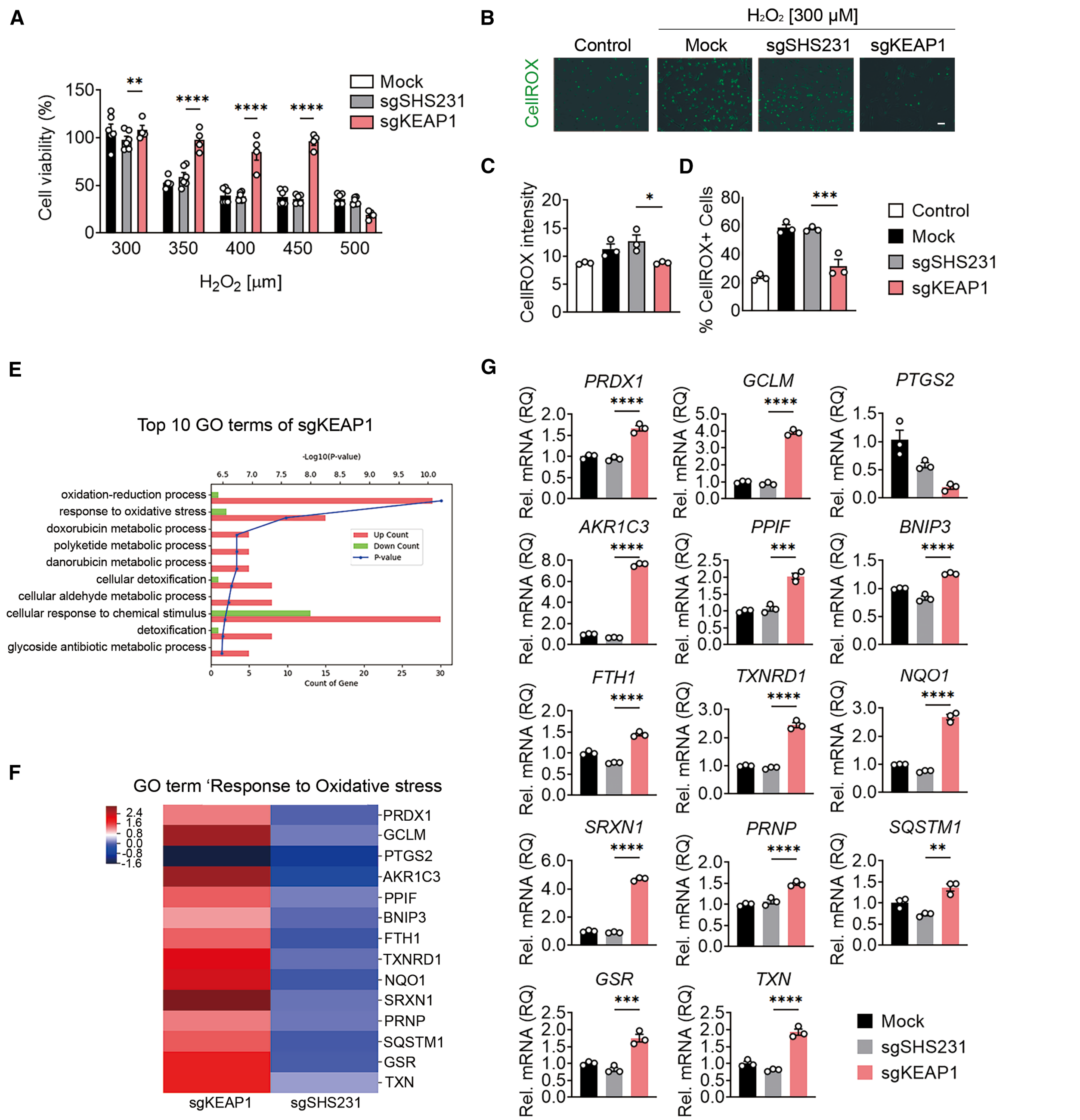

Figure 2. KEAP1 disruption enhances MSC resistance to oxidative stress and activates antioxidant gene expression

(A) Cell viability of mock, sgSHS231, or sgKEAP1 after exposure to increasing concentrations of H_2O_2 (300–500 μM) for 24 h, measured by CCK-8 assay (two-way ANOVA with Tukey's post hoc test; ***p* < 0.01; ****p* < 0.0001). (B–D) ROS levels were evaluated using CellROX staining after treatment with 300 μM H_2O_2 for 2 h. Representative fluorescence images (B), quantification of CellROX mean fluorescence intensity (C), and percentage of CellROX$^+$ cells (D) are shown (one-way ANOVA with Tukey's post hoc test; **p* < 0.05; ***p* < 0.001; *n* = 3 biological replicates; scale bar, 100 μm). (E) Top 10 Gene Ontology (GO) terms upregulated in sgKEAP1 MSCs compared to mock control identified by RNA-seq (adjusted *p* < 0.05, fold change >2). (F) Heatmap of differentially expressed genes (fold change >2) within the GO category "response to oxidative stress" for sgKEAP1 and sgSHS231 versus mock control. (G) Validation of selected oxidative stress-responsive genes identified in (F) by RT-qPCR (one-way ANOVA with Tukey's post hoc test; ****p* < 0.001; *****p* < 0.0001; *n* = 3 biological replicates).

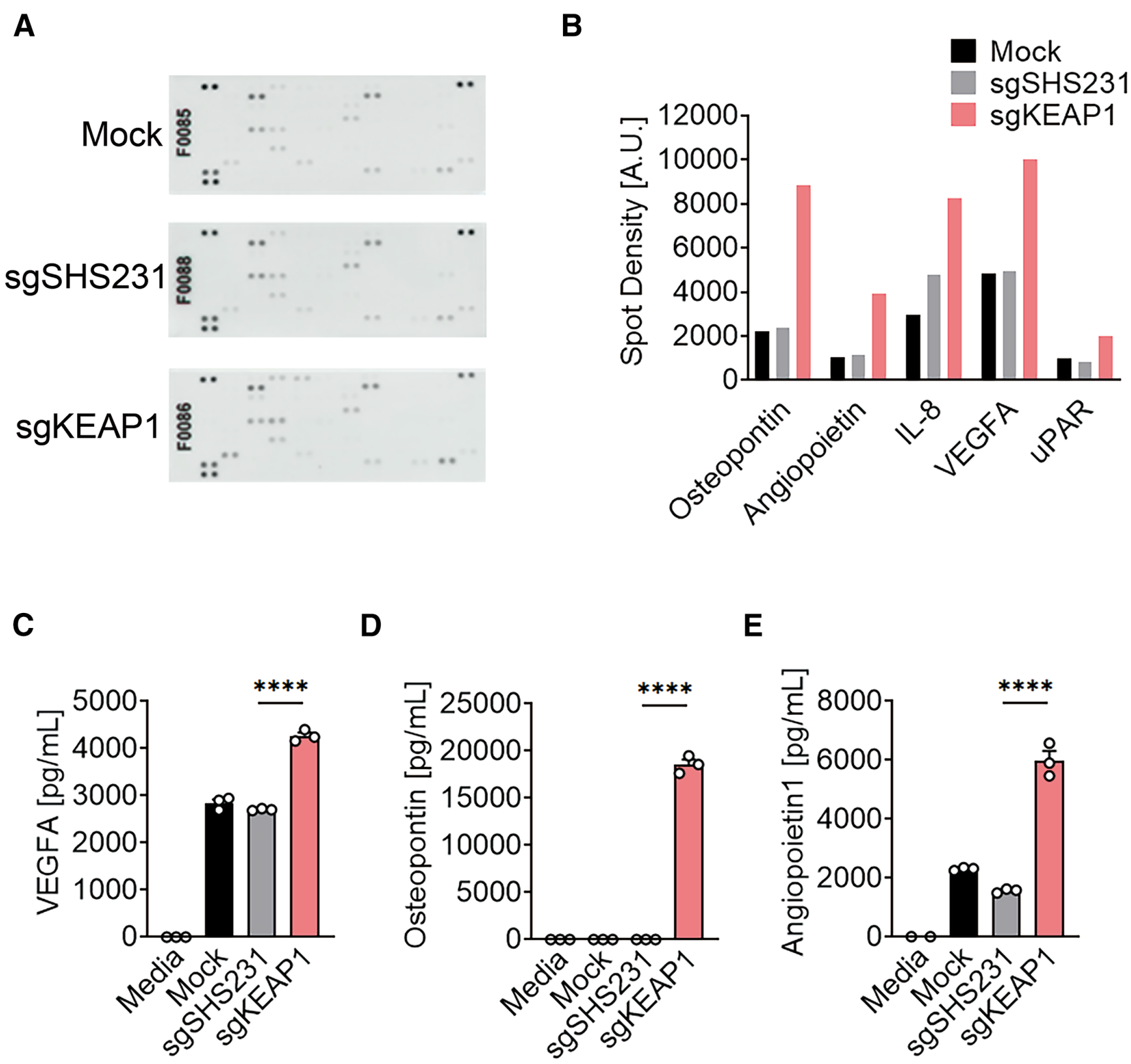

Figure 3. KEAP1 disruption enhances secretion of proangiogenic and tissue-repair cytokines

(A) Cytokine array of conditioned media collected from mock, sgSHS231, and sgKEAP1 MSCs 7 days post-gene editing. (B) Quantification of spot densities for the five most upregulated cytokines in sgKEAP1 versus controls: osteopontin, angiopoietin, IL-8, VEGFA, and uPAR. (C–E) ELISA quantification of (C) VEGFA, (D) osteopontin, and (E) angiopoietin-1 in conditioned media (one-way ANOVA with Tukey's post hoc test; ****p < 0.0001; n = 3 biological replicates).

(*Hgf*) in ischemic muscles treated with sgKEAP1 compared with control MSCs (Figures 4E–4H).

KEAP1 editing enhances angiogenesis and functional recovery in critical limb ischemia

To determine whether *KEAP1*-edited MSC improves therapeutic efficacy, we assessed blood perfusion recovery by laser Doppler perfusion imaging up to 28 days post-injection. Limbs treated with sgKEAP1 MSCs showed significantly accelerated and greater perfusion restoration compared with sgAAVS1 controls (Figures 5B and 5C). Correspondingly, sgKEAP1 MSC-treated mice exhibited lower limb necrosis scores (Figure S7). Histological analysis revealed increased density of α-smooth muscle actin-positive (α-SMA$^+$) arterioles and higher von Willebrand factor-positive (vWF$^+$) capillaries in sgKEAP1-treated tissues, whereas collagen deposition did not differ significantly (Figures 5D–5G). These findings indicate that sgKEAP1 MSCs showed enhanced arteriogenesis and tissue repair in mouse models of limb ischemia. Overall, permanent *KEAP1* disruption confers MSCs with superior survival, antioxidant defense, and paracrine support, resulting in improved vascular regeneration and functional recovery in ROS-rich ischemic environments.

DISCUSSION

In this study, we demonstrate that precise CRISPR-Cas9-mediated knockout of *KEAP1* in human MSCs leads to activation of the NRF2 antioxidant network, enhanced resistance to oxidative injury, and favored paracrine-driven regeneration in a murine model of limb ischemia. Our findings establish *KEAP1* gene editing as a functionally advantageous approach to overcome oxidative stress barriers that limit the efficacy of MSC therapy in ischemic disease. Importantly, this work provides the first comprehensive evaluation of durable NRF2 activation in clinically relevant human bone marrow-derived MSCs (hBM-MSCs) and its therapeutic impact in a CLTI model.

The oxidative milieu in ischemic tissues, including CLTI, severely restricts MSC engraftment and longevity due to excessive ROS accumulation and mitochondrial dysfunction.[11,12,42] Previous reports have shown that hypoxic or pharmacologic preconditioning can transiently boost antioxidant responses and improve cell viability, yet these effects are thought to dissipate after transplantation.[5,24,43] By contrast, our targeted disruption of *KEAP1* permanently stabilized NRF2, providing sustained activation of antioxidant defenses in the absence of exogenous DNA integration. This long-lasting genetic stabilization of NRF2 signaling distinguishes our approach from transient priming or RNAi-based methods and supports its translational suitability for durable redox enhancement. This persistent activation is supported by reduced intracellular ROS levels, elevated phosphorylated NRF2, and robust induction canonical antioxidant genes, including *PRDX1* (encodes for peroxiredoxin 1), *GSR* (glutathione-disulfide reductase), and *TXNRD1* (thioredoxin reductase 1), consistent with prior evidence that KEAP1-NRF2 signaling underpins redox homeostasis in stress conditions.[21,44,45]

The upregulated redox transcriptome in *KEAP1*-edited MSCs was accompanied by reduced intracellular ROS accumulation and improved viability under H_2O_2 challenge. This mirrors previous findings where NRF2 activation reduced oxidative apoptosis in bone marrow-derived pro-angiogenic cells in the context of hindlimb ischemia.[46] Importantly, our data extend these concepts into a translationally relevant specific *ex vivo* gene editing of MSCs. Furthermore, *in vivo* proof-of-concept study using a hindlimb ischemia model, we showed that *KEAP1*-edited MSCs not

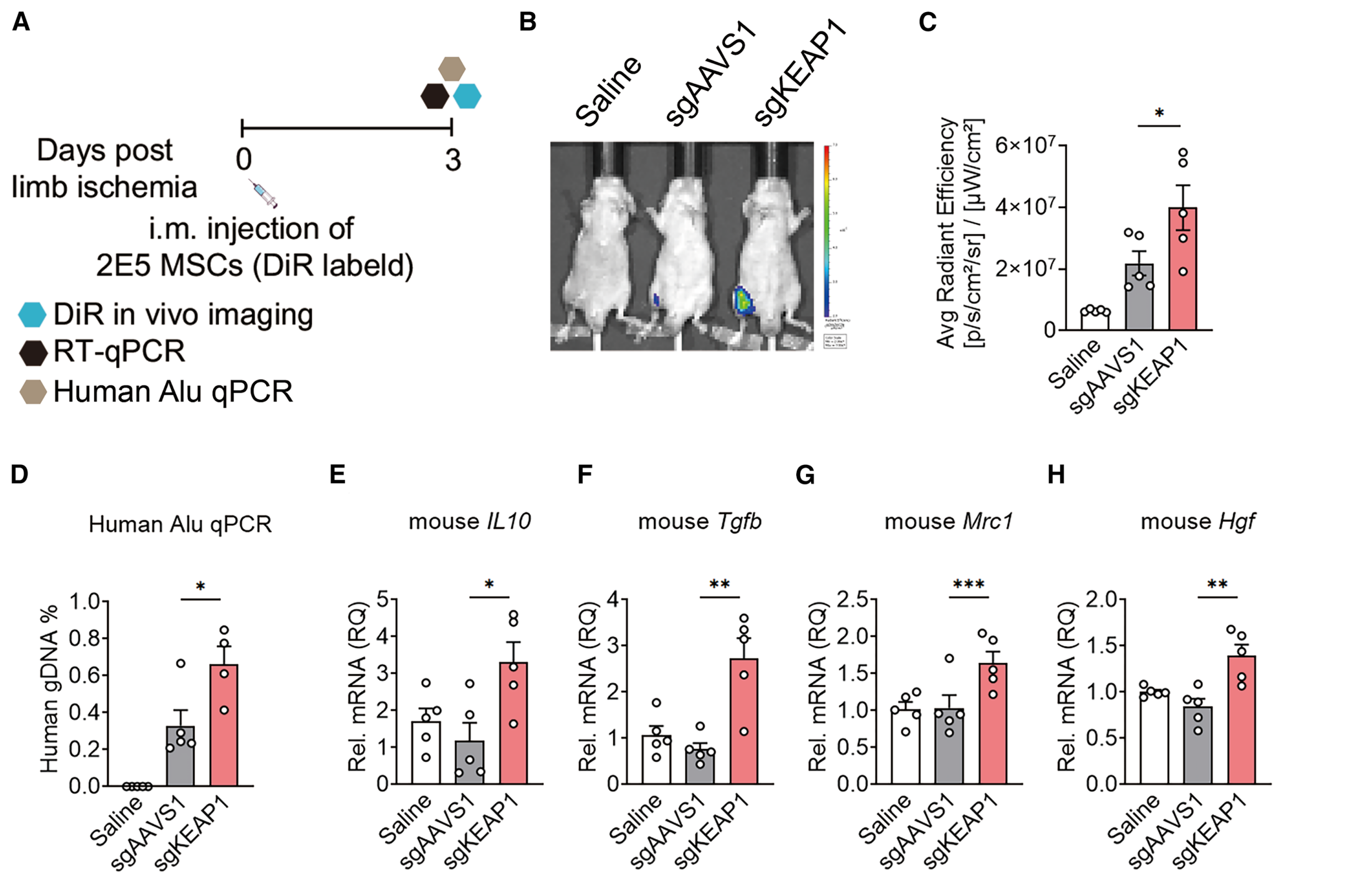

Figure 4. KEAP1 disruption enhances early MSC survival and induces pro-regenerative macrophage responses in ischemic limbs

(A) Schematic of experimental design. DiR-labeled human MSCs (3×10^5 cells); sgAAVS1 (control) or sgKEAP1 groups were intramuscularly injected into ischemic limbs of mice immediately after femoral artery ligation. Saline-injected group was used as limb ischemia-alone control. Tissues were analyzed 3 days post-injection by *in vivo* DiR imaging, RT-qPCR, and human Alu qPCR. (B and C) Representative DiR *in vivo* imaging (B) and quantification of average radiant efficiency (C) showing improved persistence of sgKEAP1 MSCs compared with controls (one-way ANOVA with Tukey's post hoc test; $p < 0.05$; $n = 5$ per group). (D) Quantification of human genomic DNA in ischemic limbs by human-specific Alu qPCR confirms enhanced engraftment of sgKEAP1 MSCs (one-way ANOVA with Tukey's post hoc test; $p < 0.05$; $n = 4$–5). (E–H) RT-qPCR analysis of mouse genes associated with M2 macrophage polarization and tissue repair (E) *Il10*, (F) *Tgfb*, (G) *Mrc1*, and (H) *Hgf* (one-way ANOVA with Tukey's post hoc test; $^*p < 0.05$; $^{**}p < 0.01$; $^{***}p < 0.001$; $n = 5$).

only survived longer in ischemic muscle but also fostered angiogenic remodeling, evidenced by increased SMA^+ arterioles, vWF^+ capillaries, and enhanced tissue perfusion. These effects were paralleled by elevated secretion of VEGFA, osteopontin, and ANG-1, aligning with the established paracrine mechanism of MSC-mediated neovascularization.[34–39,47,48]

At the mechanistic level, *KEAP1* knockout reprogrammed the MSC secretome toward cytoprotective and pro-regenerative signaling. Transcriptomic analysis revealed enrichment of pathways related to oxidative stress response and metabolic adaptation, while secretome profiling indicated increased release of immunomodulatory cytokines associated with M2 macrophage polarization. These findings resonate with recent discoveries that NRF2 activation promotes anti-inflammatory macrophage phenotypes and enhances tissue repair.[49–51] Consistent with this, ischemic limbs treated with *KEAP1*-edited MSCs displayed upregulation of host *Il10*, *Mrc1*, and *Tgfb*, suggesting paracrine modulation of the local immune microenvironment.

Our results also highlight the therapeutic advantage of gene editing over conventional priming or viral modification strategies. While RNAi-based *KEAP1* silencing can transiently increase NRF2 activity, they are limited by transient effects and off-target silencing risks.[52,53] Furthermore, retroviral or lentiviral overexpression of antioxidant genes introduces concerns about random genomic integration and transcriptional instability.[54,55] By contrast, CRISPR-Cas9 editing enables site-specific, integration-free modification with long-term functional outcomes, as previously demonstrated in edited hematopoietic stem cells.[56,57] Our work represents the first application of this strategy to enhance redox resilience in human MSCs for vascular regenerative therapy.

Given that loss-of-function mutations in *KEAP1* are reported in several malignancies, largely due to the hyperactivation of NRF2-mediated metabolic reprogramming,[58,59] the potential tumorigenicity of *KEAP1* ablation in therapeutic MSCs warrants careful consideration. In our study, *KEAP1*-edited MSCs exhibited no evidence of transformation or abnormal proliferation in long-term culture (>8 passages

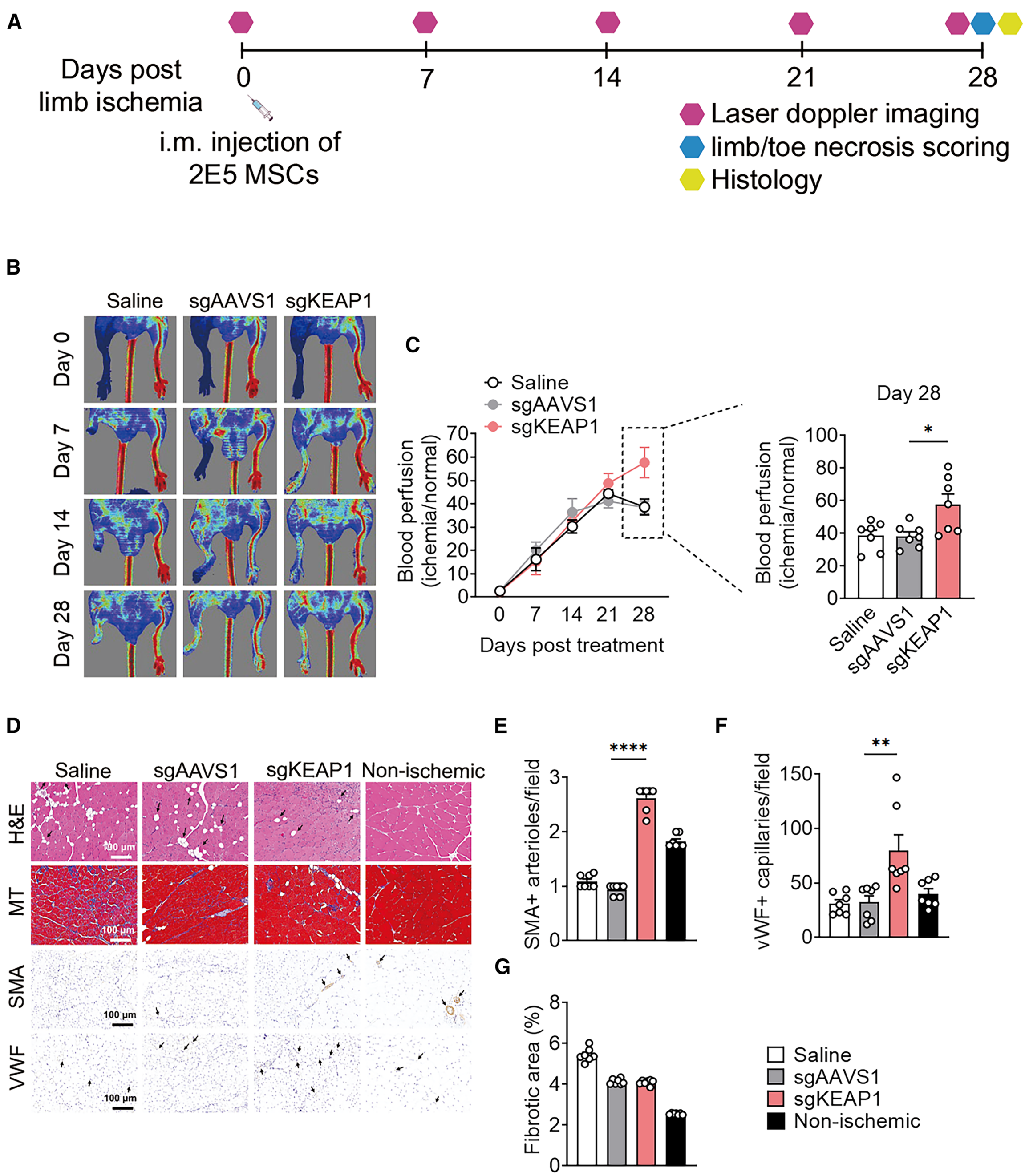

Figure 5. KEAP1-edited MSCs enhance perfusion recovery and arteriogenesis in a mouse model of critical limb ischemia

(A) Experimental timeline showing induction of unilateral hindlimb ischemia, followed by intramuscular injection of 2×10^5 MSCs (sgAAVS1 or sgKEAP1) or saline at day 0. Laser Doppler perfusion imaging (LDPI) was performed at days 0, 7, 14, 21, and 28, followed by limb necrosis scoring and histological analyses at day 28. (B) Representative LDPI pseudocolor images showing blood perfusion in ischemic (left) and contralateral (right) limbs at indicated time points. (C) Quantification of perfusion ratio (ischemic/normal limb) over time. Right: perfusion ratio at day 28 (one-way ANOVA Tukey post hoc test; $p < 0.05$; $n = 7$ mice per group). (D) Representative histological sections of ischemic gastrocnemius muscle at day 28 stained with H&E (arrows indicate fat deposition), Masson's trichrome (MT), α-SMA (arrows indicate arterioles), and vWF (arrows indicate capillaries). Scale bars, 100 μm. (E–G) Quantification of SMA^+ arterioles (E) and vWF^+ capillaries (F) per field and percentage of fibrotic area (G) ($n = 10$ fields for E and F and 30 for G per muscle section from seven mice per group; each dot represents averaged value across the fields for E and F and area-based metric per field for G; one-way ANOVA Tukey post hoc test; $^{**}p < 0.01$; $^{****}p < 0.0001$).

post-editing). Edited cells maintained adherence to plastic culture flask, stable growth kinetics, and preserved MSC surface marker profiles ($CD73^{+}CD90^{+}CD105^{+}CD45^{-}CD34^{-}$), with no spontaneous colony formation or loss of contact inhibition. In addition, although sustained NRF2 activation can potentially alter metabolic and cytoprotective pathways,[58,59] our KEAP1-edited MSCs did not show upregulation of oncogenic or cell-cycle-related genes. Furthermore, *in vivo* assessment revealed that although sgKEAP1 MSCs survived longer than control cells within ischemic muscle, DiR fluorescence and human *Alu* DNA quantification both declined to undetectable levels at the end stage of the experiment, indicating an absence of persistent engraftment or ectopic expansion. Histological analyses of injected tissues showed no teratoma-like structures or aberrant cellular proliferation. These findings align with prior studies demonstrating that KEAP1 deletion in different stem cells enhances antioxidant defense without reported malignant potential under non-oncogenic conditions.[26,60] Our data support that targeted *KEAP1* knockout in MSCs, when confined to transiently proliferative, non-immortalized primary cells, does not induce tumorigenic transformation but instead enhances redox resilience within physiological bounds, supporting its translational potential.

Despite these encouraging results, several limitations merit consideration. First, while our Digenome-seq and targeted deep sequencing confirmed minimal off-target editing, long-term genomic stability and epigenetic consequences of KEAP1 loss require further evaluation. Second, to focus on confirming the beneficial effects of KEAP1-edited MSCs, we utilized nude mice with partially impaired immune systems for modeling limb ischemia. Utilizing an immunocompetent model will provide more accurate data for translation to humans. Additionally, expanding preclinical validation across other ROS-dominant ischemic contexts such as myocardial infarction and diabetic wounds would further substantiate the generalizability of this approach.

While our findings support the therapeutic potential of *KEAP1*-edited MSCs, translational challenges remain. Large-scale Good Manufacturing Practice-compatible manufacturing and genome editing of human MSCs require stringent validation to ensure genomic stability, batch-to-batch consistency, and absence of off-target events. Furthermore, optimization of delivery routes and dosing regimens will be critical to balance efficacy and biodistribution. In future studies, evaluating these aspects using scalable editing platforms and clinically compliant culture systems will advance gene-edited MSCs toward translational readiness.

Collectively, our findings underscore that *KEAP1* knockout confers a dual benefit: intrinsic cytoprotection through NRF2 activation and extrinsic enhancement of regenerative signaling. These effects culminate in improved MSC survival, angiogenesis, and muscle tissue regeneration in limb ischemia. Given that oxidative stress also hampers cell therapy outcomes in myocardial infarction, stroke, and diabetic wounds, the same strategy could be broadly applicable across ischemia-related pathologies.

MATERIALS AND METHODS

Design and validation of sgRNAs targeting *KEAP1*

sgRNAs targeting human *KEAP1* exon 2–3 were designed using CRISPR RGEN Tools Cas-designer (http://www.rgenome.net/cas-designer/). Fifteen candidate sgRNAs (sgKEAP1_1–15) were synthesized by *in vitro* transcription using T7 polymerase (New England Biolabs) according to the manufacturer's protocol. A non-targeting control sgRNA targeting *SHS231* or *AAVS1* locus served as reference (sgSHS231 or sgAAVS1, respectively). MSCs were nucleofected with Cas9-gRNA RNP complexes using the P3 Primary Cell 4D-Nucleofector X Kit (Lonza, program EW-104).

hBM-MSC culture

hBM-MSCs were obtained from commercially available sources (Lonza; Table S6). Cells were cultured in minimum essential medium α (MEMα; Gibco) supplemented with 10% fetal bovine serum (FBS; Gibco), gentamicin (Gibco, 5 μg/mL), and human fibroblast growth factor (Gibco, 1 ng/mL) at 37°C under 5% CO_2. Cells at passages 4–6 were used for all experiments. For the transfection of CRISPR-Cas9 components, RNP complexes were formed by Cas9 protein (Aldevron) with sgRNA at room temperature for 15 min. MSCs were nucleofected with RNP complexes using the P1 Primary Cell 4D-Nucleofector X Kit (Lonza, program EW-104). To evaluate cell viability and proliferation, the number of cells before and after each passage was measured using trypan blue staining. The growth rate was calculated by dividing the harvested cell number by the seeding cell number, and the logarithm base 2 of this value was defined as the population doubling level (PDL). The PDT was calculated by dividing the PDL by the culture duration.

Targeted deep sequencing and indel quantification

Genomic DNA (gDNA) was extracted at 72 h or specified time post-transfection using the DNeasy Blood & Tissue Kit (QIAGEN). Targeted deep sequencing was performed as before.[61] Briefly, the on-target region was PCR amplified from gDNA extracted from transfected cells using Phusion polymerase (New England Biolabs). The resulting PCR amplicons were then subjected to paired-end deep sequencing using Mi-Seq (Illumina). Data from deep sequencing were analyzed using the online Cas-Analyzer tool (www.rgenome.net). Indels in the region 3 bp upstream from the PAM sequence were considered to be mutations resulting from Cas9.

In silico identification of off-target sites

Potential off-target sites with ≤ 2 mismatches were predicted *in silico* using an online tool (www.rgenome.net).[62]

Digenome-seq

Digenome-seq was performed as previously described.[61] Briefly, gDNA was purified from HEK293T cells and mixed with the RNP complex and incubated at 37°C for 8 h. Digested gDNA was treated with RNAse A and purified again with a DNeasy Tissue Kit (QIAGEN). Digested gDNA was fragmented using the Covaris system, which was then ligated with adapters to produce libraries. These

libraries were then subjected to whole-genome sequencing using a HiSeq X Ten Sequencer (Illumina) at a sequencing depth of 30–40× (Macrogen). An *in vitro* DNA cleavage score was calculated at each nucleotide position across the genome with a DNA cleavage scoring system described previously.[30]

Verification of off-target sites

Ten identified potential off-target candidate loci from *in silico* and Digenome-seq analyses were PCR amplified from gDNA extracted from either mock or sgKEAP1-transfected MSCs and subjected to targeted deep sequencing. Indel frequencies were quantified with 0.01% as the lowest detectable level (floor).

RT-qPCR of MSCs

Total RNA was isolated using RNeasy Mini kit (QIAGEN) following the manufacturer's instruction with the High-Capacity cDNA Reverse Transcription Kit (Applied Biosystems). RT-qPCR was performed with the SYBR Green Master Mix (Thermo Fisher) protocol. Relative expression was calculated by the ΔΔCt method normalized to *GAPDH* (glyceraldehyde 3-phosphate dehydrogenase). The primers used in this study are shown in Table S5.

Western blotting

Cells were lysed in radioimmunoprecipitation assay buffer (Thermo Fisher) containing protease and phosphatase inhibitors (Thermo Fisher). Equal protein (20 μg) was separated by SDS-PAGE and transferred to polyvinylidene fluoride membranes. The primary antibodies were anti-KEAP1 (Cell Signaling Technology, catalog no. 4678), anti-phospho-NRF2 (Abcam, catalog no. ab76026), anti-NRF2 (Cell Signaling Technology, catalog no. 12721), and β-actin (Cell Signaling Technology, catalog no. 4970). Bands were visualized using horseradish peroxidase-conjugated secondary antibodies and enhanced chemiluminescence reagent (Thermo Fisher).

Flow cytometry

Cell staining was performed at 4°C in PBS supplemented with 2% FBS unless otherwise indicated. Antibodies and reagents used for flow cytometry and functional studies were as follows: PE (phycoerythrin) Mouse Anti-Human CD34 antibody (BioLegend, catalog no. 343506), APC (allophycocyanin) Mouse Anti-Human CD45 antibody (BioLegend, catalog no. 368512), APC/Cy7 Anti-Human CD73 antibody (BioLegend, catalog no. 344022), PE anti-human CD73 antibody (BioLegend, catalog no. 344004), fluorescein isothiocyanate anti-human CD90 antibody (BioLegend, catalog no. 328108), APC anti-human CD44 antibody (BioLegend, catalog no. 397506), and APC anti-human human leukocyte antigen-DR isotype antibody (BioLegend, catalog no. 361610). Data were collected on an Attune NxT Acoustic Focusing Cytometer (Thermo Scientific) and analyzed using FlowJo.

Oxidative stress assays

Equal cell numbers were seeded in each well and allowed to adhere uniformly for 24 h before treatment, with visual confluency confirmation to minimize variability in initial cell density. MSCs were exposed to 200–500 μM H_2O_2 diluted in culture media immediately before use for 24 h. Viability was quantified using CCK-8 (Dojindo). Absorbance was measured at 450 nm and normalized to baseline and untreated controls within each experimental group. Intracellular ROS was measured using CellROX Green (Thermo Fisher, 5 μM, 30 min at 37°C). Fluorescence intensity and percentage $CellROX^+$ cells were quantified using ImageJ from 10 random fields (≥300 cells per condition) acquired at 20× magnification. To assess oxidative stress-induced cell death, cells were treated with Annexin V Apoptosis Detection Kit with 7-AAD (BioLegend) according to the manufacturer's protocol, and data were collected on an Attune NxT acoustic Focusing Cytometer (Thermo Scientific). Apoptotic cells were defined as annexin V^+/7-AAD^- (early apoptosis) and annexin V^+/7-AAD^+ (late apoptosis/necrosis).

RNA-seq and bioinformatic analysis

RNA-seq was performed at ebiogen (Seoul, Republic of Korea). Briefly, total RNA was isolated using RNeasy Mini kit (QIAGEN). RNA quality was assessed by a Agilent 2100 bioanalyzer using the RNA 6000 Nano Chip (Agilent Technologies), and RNA quantification was performed using an ND-2000 Spectrophotometer (Thermo Fisher). For control and test RNAs, the construction of the library was performed using QuantSeq 3′ mRNA-Seq Library Prep Kit (Lexogen) according to the manufacturer's instructions. In brief, each 500-ng total RNA was prepared and an oligo(dT) primer containing an Illumina-compatible sequence at its 5′ end was hybridized to the RNA, and reverse transcription was performed. After degradation of the RNA template, second-strand synthesis was initiated by a random primer containing an Illumina-compatible linker sequence at its 5′ end. The double-stranded library was purified by using magnetic beads to remove all reaction components. The library was amplified to add the complete adapter sequences required for cluster generation. The finished library is purified from PCR components. High-throughput sequencing was performed as single-end 75 sequencing using NextSeq 500 (Illumina). QuantSeq 3′ mRNA-seq reads were aligned using Bowtie2.[63] Bowtie2 indices were either generated from the genome assembly sequence or the representative transcript sequences for aligning to the genome and transcriptome. The alignment file was used for assembling transcripts, estimating their abundances, and detecting differential expressions of genes. Differentially expressed genes were determined based on counts from unique and multiple alignments using coverage in BEDtools.[64] The read count data were processed based on the TMM+CPM normalization method using EdgeR within R using Bioconductor.[65] Functional enrichment analysis was performed using GO Biological Process terms. RNA-seq was performed with three biological replicates per group (n = 3).

Cytokine array and ELISA

Conditioned media were collected from MSCs at 80%–90% confluence after 48-h incubation in serum-free MEMα, followed by centrifugation (500 × g, 5 min) to remove debris, and supernatants were tested for cytokines using Human XL cytokine array (R&D Systems, catalog no. ARY022). The procedure was performed according to the

manufacturer's instructions. Then, the membranes were developed, and the dots were analyzed using an LI-COR Odyssey Infrared Imaging System. Spot density was quantified with ImageJ and normalized to internal standards.

Secreted VEGFA, osteopontin, and ANG-1 were quantified using commercial ELISA kits (R&D Systems) following the manufacturer's instructions.

Murine model of CLTI

All animal procedures were conducted in accordance with institutional guidelines and were approved by the Institutional Animal Care and Use Committee of the Yonsei University Health System (permit no. 2022-0038). Mice were housed in pathogen-free facilities under standard conditions, and all efforts were made to minimize suffering, including perioperative analgesia and humane endpoints for animals exhibiting severe limb necrosis or distress. Hindlimb ischemia was induced in anesthetized athymic female mice (Balb/c-nu, 7 weeks old, Orient Bio) as previously described.[66–68] After skin incision, the left iliac and femoral arteries were permanently ligated using a 6-0 prolene suture (Ethicon). Specifically, to prevent excessive bleeding, the external iliac artery was ligated with a suture, and the femoral artery was excised from its origin at the external iliac artery down to the saphenous artery. Sutures were placed at both ends of the femoral artery adjacent to the external iliac artery and the distal saphenous artery near the ankle. The boundary of the femoral artery excision sites were marked with a non-absorbable suture at the time of surgery to provide a consistent anatomical reference point during tissue harvesting and sectioning. Immediately after ligation of the arteries, mice received an intramuscular injection of 2×10^5 MSCs (mock, sgAAVS1, or sgKEAP1) suspended in 50 μL saline into the ischemic gastrocnemius. All animal studies were performed in a blinded manner. The status of the lower extremity of the left limb on day 28 was evaluated to determine its physiological status score. Amputation of the lower extremity was designated as limb loss, and rotten skin and muscle of the left limb were designated as necrosis. The blood perfusion in ischemic hindlimbs was monitored by serial scanning using a laser Doppler imaging system (MoorLDI2-HIR High Resolution, Moor Instruments) on days 0, 7, 14, 21, and 28 after treatment. Blood flow was quantified using digital color-coded images from the knee joint region to the toe region, and the perfusion rate was calculated as the ratio of ischemic limb to normal limb. All experimental groups were initially designed with $n = 10$ animals per group. During the 28-day study period, complete limb loss occurred in a subset of animals, and the final sample size for perfusion analysis was therefore determined based on the group with the highest exclusion rate, resulting in $n = 7$ animals per group. To enable balanced group-wise comparison and to avoid bias arising from unequal sample sizes, in groups with more than seven eligible animals, randomization was performed to select seven animals each for analysis. Randomization was performed using the RAND function in Microsoft Excel. Four weeks after transplantation, the mice were sacrificed for histological analysis.

In vivo imaging and cell persistence

MSCs were labeled with DiR (Thermo Fisher) before transplantation. Bioluminescent imaging was performed using an IVIS Spectrum system at day 3 post-injection. Human cell persistence was quantified from ischemic tissue by human *Alu* qPCR normalized to mouse *Gapdh*. qPCR was conducted to measure the level of human-specific *Alu* genes from a 33-ng DNA specimen in 10 μL PCR reagent mixture containing 200 nM primers and 200 nM probe. The primers used in this study were as follows: human Alu F: 5′-GGTGAAACCCCGTCTCTACT-3′ and human Alu R: 5′-GGTTCAAGCGATTCTCCTGC-3′, and the probe was 5'-CGCCCGGCTAATTTTTGTAT-3′. A standard curve was generated by serial dilution of human gDNA from hMSCs into DNA from mice tissue (the total DNA amount was kept constant at 33 ng). According to the standard curve, the percentage of human DNA in 33 ng total DNA from each organ or tissue was calculated.[69]

RT-qPCR of mouse limb tissues

Total RNA was isolated using the RNeasy Fibrous Tissue Mini Kit (QIAGEN, catalog no. 74704) following the manufacturer's instruction with the High-Capacity cDNA Reverse Transcription Kit (Applied Biosystems). RT-qPCR was performed with SYBR Green Master Mix (Thermo Scientific). Relative expression was calculated by the ΔΔCt method normalized to *Gapdh*. The primers used in this study are shown in Table S5.

Histology and immunostaining

Histologic analyses were performed only on ischemic limbs that remained intact. Animals that experienced complete limb amputation before tissue harvest were excluded. Harvested ischemic muscles were fixed with 4% paraformaldehyde (Sigma) and paraffin embedded. Serial sections were obtained from comparable anatomical locations of the ischemic limbs. Specifically, tissue sections were collected at equivalent distances from the femoral artery excision sites across all animals. The sections were stained with H&E and Masson's trichrome. The fibrotic area was quantified as the percentage of collagen-stained area in Masson's trichrome-stained images using Fiji (National Institutes of Health) and reported as an area-based metric. The serial sections were immunofluorescently stained for capillaries and arterioles using anti-vWF (MilliporeSigma, catalog no. AB7356, 1:100 dilution) and anti-α-SMA antibodies (Santa Cruz Biotechnology, catalog no. sc-53142, 1:100 dilution) following standard histological procedures. The densities of capillaries and arterioles were determined by counting vWF^+ capillaries and $\alpha\text{-SMA}^+$ microvessels, respectively, and arteriole size was measured using Fiji software based on the fluorescent images of $\alpha\text{-SMA}^+$ microvessels. Analyses were performed using 10–30 microscopic fields per limb were quantified from seven mice per group. For the measurements of capillaries and arterioles, vessel counts were performed per microscopic field, and average mean values were reported.

Statistical analysis

All experiments were performed with at least three independent biological replicates unless otherwise indicated. Data were expressed as

mean ± SEM. Statistical analyses were performed using GraphPad Prism. Comparisons among multiple groups were made using one-way ANOVA with Tukey's post hoc test or two-way ANOVA for multi-factor experiments. Prior to statistical analyses, all datasets were tested for normality using the Shapiro-Wilk test. A $p < 0.05$ was considered statistically significant.

DATA AND CODE AVAILABILITY

The datasets generated or analyzed during the present study are available from the corresponding author on reasonable request.

ACKNOWLEDGMENTS

This work was supported by the Korea Health Industry Development Institute, funded by the Ministry of Health & Welfare, Republic of Korea (RS-2025-16068430), Alchemist Project 2410012609 (20012378, Development of Meta Soft Organ Module Manufacturing Technology without Immunity Rejection and Module Assembly Robot System) funded by the Ministry of Trade, Industry & Energy, Korea, the National Research Foundation of Korea grant funded by the Ministry of Science and ICT, Republic of Korea (RS-2021-NR059722), Samsung Medical Center grant no. SMO126023 and the 63Research Fund, Sungkyunkwan University, 2026.

AUTHOR CONTRIBUTIONS

E.J.S., Y.C., E.J.J., K.-i.L., K.-j.L., Y.J.S., M.J.S., and S.K. performed the experiments and interpreted the data. E.J.S. and J.Y.L. wrote the manuscript. J.Y.L. and S.-W.C. supervised the project and revised the manuscript.

DECLARATION OF INTERESTS

E.J.S., K.-i.L., K.-j.L., and M.J.S. are currently employed by ToolGen, Inc. E.J.J. and S.-W.C. are employed by Cellartgen, Inc. There are patent documents related to this work.

SUPPLEMENTAL INFORMATION

Supplemental information can be found online at https://doi.org/10.1016/j.ymthe.2026.03.005.

REFERENCES

1. Shu, J., and Santulli, G. (2018). Update on peripheral artery disease: Epidemiology and evidence-based facts. Atherosclerosis *275*, 379–381. https://doi.org/10.1016/j.atherosclerosis.2018.05.033.

2. Daher, G., Upadhyay, S., and Li, J. (2025). Chronic Limb-Threatening Ischemia: A Comprehensive Review Paper. Interv. Cardiol. Clin. *14*, 257–272. https://doi.org/10.1016/j.iccl.2024.11.011.

3. Beegle, J., Lakatos, K., Kalomoiris, S., Stewart, H., Isseroff, R.R., Nolta, J.A., and Fierro, F.A. (2015). Hypoxic preconditioning of mesenchymal stromal cells induces metabolic changes, enhances survival, and promotes cell retention in vivo. Stem Cells *33*, 1818–1828. https://doi.org/10.1002/stem.1976.

4. De Palma, S.T., Hermans, E.C., Shamorkina, T.M., Trayford, C., van Rijt, S., Heck, A.J.R., Nijboer, C.H.A., and de Theije, C.G.M. (2025). Hypoxic Preconditioning Enhances the Potential of Mesenchymal Stem Cells to Treat Neonatal Hypoxic-Ischemic Brain Injury. Stroke *56*, 1872–1882. https://doi.org/10.1161/STROKEAHA.124.048964.

5. Peterson, K.M., Aly, A., Lerman, A., Lerman, L.O., and Rodriguez-Porcel, M. (2011). Improved survival of mesenchymal stromal cell after hypoxia preconditioning: role of oxidative stress. Life Sci. *88*, 65–73. https://doi.org/10.1016/j.lfs.2010.10.023.

6. Dopheide, J.F., Doppler, C., Scheer, M., Obst, V., Radmacher, M.C., Radsak, M.P., Gori, T., Warnholtz, A., Fottner, C., Münzel, T., et al. (2013). Critical limb ischaemia is characterised by an increased production of whole blood reactive oxygen species and expression of TREM-1 on neutrophils. Atherosclerosis *229*, 396–403. https://doi.org/10.1016/j.atherosclerosis.2013.05.029.

7. Caicedo, D., Devesa, P., Arce, V.M., Requena, J., and Devesa, J. (2018). Chronic limb-threatening ischemia could benefit from growth hormone therapy for wound healing and limb salvage. Ther. Adv. Cardiovasc. Dis. *12*, 53–72. https://doi.org/10.1177/1753944717745494.

8. Lin, Y.C., Shih, J.Y., Lin, Y.W., Niu, K.C., Hong, C.S., Chen, Z.C., Pan, S.C., Chang, T.Y., Kan, W.C., and Chang, W.T. (2024). Hyperbaric Oxygen Therapy Improved Neovascularisation Following Limb Ischaemia-The Role of ROS Mitigation. J. Cell. Mol. Med. *28*, e70310. https://doi.org/10.1111/jcmm.70310.

9. Rocha-Ferreira, E., Kelen, D., Faulkner, S., Broad, K.D., Chandrasekaran, M., Kerenyi, Á., Kato, T., Bainbridge, A., Golay, X., Sullivan, M., et al. (2017). Systemic pro-inflammatory cytokine status following therapeutic hypothermia in a piglet hypoxia-ischemia model. J. Neuroinflammation *14*, 44. https://doi.org/10.1186/s12974-017-0821-x.

10. Zheng, D., Liu, J., Piao, H., Zhu, Z., Wei, R., and Liu, K. (2022). ROS-triggered endothelial cell death mechanisms: Focus on pyroptosis, parthanatos, and ferroptosis. Front. Immunol. *13*, 1039241. https://doi.org/10.3389/fimmu.2022.1039241.

11. Signorelli, S.S., Vanella, L., Abraham, N.G., Scuto, S., Marino, E., and Rocic, P. (2020). Pathophysiology of chronic peripheral ischemia: new perspectives. Ther. Adv. Chronic Dis. *11*, 2040622319894466. https://doi.org/10.1177/2040622319894466.

12. Gu, Y., Rampin, A., Alvino, V.V., Spinetti, G., and Madeddu, P. (2021). Cell Therapy for Critical Limb Ischemia: Advantages, Limitations, and New Perspectives for Treatment of Patients with Critical Diabetic Vasculopathy. Curr. Diab. Rep. *21*, 11. https://doi.org/10.1007/s11892-021-01378-4.

13. Caplan, A.I., and Correa, D. (2011). The MSC: an injury drugstore. Cell Stem Cell *9*, 11–15. https://doi.org/10.1016/j.stem.2011.06.008.

14. Lozano Navarro, L.V., Chen, X., Giratá Viviescas, L.T., Ardila-Roa, A.K., Luna-Gonzalez, M.L., Sossa, C.L., and Arango-Rodríguez, M.L. (2022). Mesenchymal stem cells for critical limb ischemia: their function, mechanism, and therapeutic potential. Stem Cell Res. Ther. *13*, 345. https://doi.org/10.1186/s13287-022-03043-3.

15. Hu, C., Zhao, L., Peng, C., and Li, L. (2018). Regulation of the mitochondrial reactive oxygen species: Strategies to control mesenchymal stem cell fates ex vivo and in vivo. J. Cell. Mol. Med. *22*, 5196–5207. https://doi.org/10.1111/jcmm.13835.

16. Denu, R.A., and Hematti, P. (2016). Effects of Oxidative Stress on Mesenchymal Stem Cell Biology. Oxid. Med. Cell. Longev. *2016*, 2989076. https://doi.org/10.1155/2016/2989076.

17. Mohammadzadeh, M., Halabian, R., Gharehbaghian, A., Amirizadeh, N., Jahanian-Najafabadi, A., Roushandeh, A.M., and Roudkenar, M.H. (2012). Nrf-2 overexpression in mesenchymal stem cells reduces oxidative stress-induced apoptosis and cytotoxicity. Cell Stress Chaperones *17*, 553–565. https://doi.org/10.1007/s12192-012-0331-9.

18. Yuan, Z., Zhang, J., Huang, Y., Zhang, Y., Liu, W., Wang, G., Zhang, Q., Wang, G., Yang, Y., Li, H., and Chen, G. (2017). NRF2 overexpression in mesenchymal stem cells induces stem-cell marker expression and enhances osteoblastic differentiation. Biochem. Biophys. Res. Commun. *491*, 228–235. https://doi.org/10.1016/j.bbrc.2017.07.083.

19. Zhaleh, F., Amiri, F., Mohammadzadeh-Vardin, M., Bahadori, M., Harati, M.D., Roudkenar, M.H., and Saki, S. (2016). Nuclear factor erythroid-2 related factor 2 overexpressed mesenchymal stem cells transplantation, improves renal function, decreases injuries markers and increases repair markers in glycerol-induced Acute kidney injury rats. Iran. J. Basic Med. Sci. *19*, 323–329.

20. Chen, F., Che, Z., Liu, Y., Luo, P., Xiao, L., Song, Y., Wang, C., Dong, Z., Li, M., Tipoe, G.L., et al. (2024). Invigorating human MSCs for transplantation therapy via Nrf2/DKK1 co-stimulation in an acute-on-chronic liver failure mouse model. Gastroenterol. Rep. *12*, goae016. https://doi.org/10.1093/gastro/goae016.

21. Rabbani, P.S., Soares, M.A., Hameedi, S.G., Kadle, R.L., Mubasher, A., Kowzun, M., and Ceradini, D.J. (2019). Dysregulation of Nrf2/Keap1 Redox Pathway in Diabetes Affects Multipotency of Stromal Cells. Diabetes *68*, 141–155. https://doi.org/10.2337/db18-0232.

22. Itoh, K., Wakabayashi, N., Katoh, Y., Ishii, T., Igarashi, K., Engel, J.D., and Yamamoto, M. (1999). Keap1 represses nuclear activation of antioxidant responsive elements by Nrf2 through binding to the amino-terminal Neh2 domain. Genes Dev. *13*, 76–86. https://doi.org/10.1101/gad.13.1.76.

23. McMahon, M., Itoh, K., Yamamoto, M., and Hayes, J.D. (2003). Keap1-dependent proteasomal degradation of transcription factor Nrf2 contributes to the negative regulation of antioxidant response element-driven gene expression. J. Biol. Chem. *278*, 21592–21600. https://doi.org/10.1074/jbc.M300931200.

24. Wang, S., Zhang, C., Niyazi, S., Zheng, L., Li, J., Zhang, W., Xu, M., Rong, R., Yang, C., and Zhu, T. (2017). A novel cytoprotective peptide protects mesenchymal stem cells against mitochondrial dysfunction and apoptosis induced by starvation via Nrf2/Sirt3/FoxO3a pathway. J. Transl. Med. *15*, 33. https://doi.org/10.1186/s12967-017-1144-5.

25. Puig-Serra, P., Hinckley-Boned, A., Tristán-Manzano, M., Rio, P., Torres-Ruiz, R., Rodriguez-Perales, S., and Martín, F. (2025). Risk and Benefit Assessment of Gene Therapy with Lentiviral Vectors and Hematopoietic Stem Cells: The Skysona Case. Hum. Gene Ther. *36*, 1159–1172. https://doi.org/10.1177/10430342251372474.

26. Hu, Y., Liu, S., and Zhu, B.M. (2019). CRISPR/Cas9-Induced Loss of Keap1 Enhances Anti-oxidation in Rat Adipose-Derived Mesenchymal Stem Cells. Front. Neurol. *10*, 1311. https://doi.org/10.3389/fneur.2019.01311.

27. Pellenz, S., Phelps, M., Tang, W., Hovde, B.T., Sinit, R.B., Fu, W., Li, H., Chen, E., and Monnat, R.J., Jr. (2019). New Human Chromosomal Sites with "Safe Harbor" Potential for Targeted Transgene Insertion. Hum. Gene Ther. *30*, 814–828. https://doi.org/10.1089/hum.2018.169.

28. Kotin, R.M., Linden, R.M., and Berns, K.I. (1992). Characterization of a preferred site on human chromosome 19q for integration of adeno-associated virus DNA by non-homologous recombination. EMBO J. *11*, 5071–5078. https://doi.org/10.1002/j.1460-2075.1992.tb05614.x.

29. Chuai, G.H., Wang, Q.L., and Liu, Q. (2017). In Silico Meets In Vivo: Towards Computational CRISPR-Based sgRNA Design. Trends Biotechnol. *35*, 12–21. https://doi.org/10.1016/j.tibtech.2016.06.008.

30. Kim, D., Bae, S., Park, J., Kim, E., Kim, S., Yu, H.R., Hwang, J., Kim, J.I., and Kim, J.S. (2015). Digenome-seq: genome-wide profiling of CRISPR-Cas9 off-target effects in human cells. Nat. Methods *12*, 237–243. https://doi.org/10.1038/nmeth.3284.

31. Lim, J., Heo, J., Ju, H., Shin, J.W., Kim, Y., Lee, S., Yu, H.Y., Ryu, C.M., Yun, H., Song, S., et al. (2020). Glutathione dynamics determine the therapeutic efficacy of mesenchymal stem cells for graft-versus-host disease via CREB1-NRF2 pathway. Sci. Adv. *6*, eaba1334. https://doi.org/10.1126/sciadv.aba1334.

32. García-Guerrero, C.A., Fuentes, P., Araya, M.J., Djouad, F., Luz-Crawford, P., Vega-Letter, A.M., and Altamirano, C. (2024). How to enhance MSCs therapeutic properties? An insight on potentiation methods. Stem Cell Res. Ther. *15*, 331. https://doi.org/10.1186/s13287-024-03935-6.

33. Chang, C., Yan, J., Yao, Z., Zhang, C., Li, X., and Mao, H.Q. (2021). Effects of Mesenchymal Stem Cell-Derived Paracrine Signals and Their Delivery Strategies. Adv. Healthc. Mater. *10*, e2001689. https://doi.org/10.1002/adhm.202001689.

34. Masaki, I., Yonemitsu, Y., Yamashita, A., Sata, S., Tanii, M., Komori, K., Nakagawa, K., Hou, X., Nagai, Y., Hasegawa, M., et al. (2002). Angiogenic gene therapy for experimental critical limb ischemia: acceleration of limb loss by overexpression of vascular endothelial growth factor 165 but not of fibroblast growth factor-2. Circ. Res. *90*, 966–973. https://doi.org/10.1161/01.res.0000019540.41697.60.

35. van Weel, V., Deckers, M.M.L., Grimbergen, J.M., van Leuven, K.J.M., Lardenoye, J.H.P., Schlingemann, R.O., van Nieuw Amerongen, G.P., van Bockel, J.H., van Hinsbergh, V.W.M., and Quax, P.H.A. (2004). Vascular endothelial growth factor overexpression in ischemic skeletal muscle enhances myoglobin expression in vivo. Circ. Res. *95*, 58–66. https://doi.org/10.1161/01.RES.0000133247.69803.c3.

36. Duvall, C.L., Weiss, D., Robinson, S.T., Alameddine, F.M.F., Guldberg, R.E., and Taylor, W.R. (2008). The role of osteopontin in recovery from hind limb ischemia. Arterioscler. Thromb. Vasc. Biol. *28*, 290–295. https://doi.org/10.1161/ATVBAHA.107.158485.

37. Lee, G.S., Salazar, H.F., Joseph, G., Lok, Z.S.Y., Caroti, C.M., Weiss, D., Taylor, W.R., and Lyle, A.N. (2019). Osteopontin isoforms differentially promote arteriogenesis in response to ischemia via macrophage accumulation and survival. Lab. Invest. *99*, 331–345. https://doi.org/10.1038/s41374-018-0094-8.

38. Kobayashi, K., Kondo, T., Inoue, N., Aoki, M., Mizuno, M., Komori, K., Yoshida, J., and Murohara, T. (2006). Combination of in vivo angiopoietin-1 gene transfer and autologous bone marrow cell implantation for functional therapeutic angiogenesis. Arterioscler. Thromb. Vasc. Biol. *26*, 1465–1472. https://doi.org/10.1161/01.ATV.0000223865.64812.26.

39. Huang, J., Inoue, M., Hasegawa, M., Tomihara, K., Tanaka, T., Chen, J., and Hamada, H. (2009). Sendai viral vector mediated angiopoietin-1 gene transfer for experimental ischemic limb disease. Angiogenesis *12*, 243–249. https://doi.org/10.1007/s10456-009-9144-6.

40. Choi, J., Choi, W., Joo, Y., Chung, H., Kim, D., Oh, S.J., and Kim, S.H. (2021). FGF2-primed 3D spheroids producing IL-8 promote therapeutic angiogenesis in murine hindlimb ischemia. NPJ Regen. Med. *6*, 48. https://doi.org/10.1038/s41536-021-00159-7.

41. Ouriel, K., Veith, F.J., and Sasahara, A.A. (1998). A comparison of recombinant urokinase with vascular surgery as initial treatment for acute arterial occlusion of the legs. Thrombolysis or Peripheral Arterial Surgery (TOPAS) Investigators. N. Engl. J. Med. *338*, 1105–1111. https://doi.org/10.1056/NEJM199804163381603.

42. Khodayari, S., Khodayari, H., Ebrahimi-Barough, S., Khanmohammadi, M., Islam, M.S., Vesovic, M., Goodarzi, A., Mahmoodzadeh, H., Nayernia, K., Aghdami, N., and Ai, J. (2022). Stem Cell Therapy in Limb Ischemia: State-of-Art, Perspective, and Possible Impacts of Endometrial-Derived Stem Cells. Front. Cell Dev. Biol. *10*, 834754. https://doi.org/10.3389/fcell.2022.834754.

43. Watanabe, J., Yamada, M., Niibe, K., Zhang, M., Kondo, T., Ishibashi, M., and Egusa, H. (2018). Preconditioning of bone marrow-derived mesenchymal stem cells with N-acetyl-L-cysteine enhances bone regeneration via reinforced resistance to oxidative stress. Biomaterials *185*, 25–38. https://doi.org/10.1016/j.biomaterials.2018.08.055.

44. Yan, X., Fu, X., Jia, Y., Ma, X., Tao, J., Yang, T., Ma, H., Liang, X., Liu, X., Yang, J., and Wei, J. (2019). Nrf2/Keap1/ARE Signaling Mediated an Antioxidative Protection of Human Placental Mesenchymal Stem Cells of Fetal Origin in Alveolar Epithelial Cells. Oxid. Med. Cell. Longev. *2019*, 2654910. https://doi.org/10.1155/2019/2654910.

45. Dai, X., Yan, X., Wintergerst, K.A., Cai, L., Keller, B.B., and Tan, Y. (2020). Nrf2: Redox and Metabolic Regulator of Stem Cell State and Function. Trends Mol. Med. *26*, 185–200. https://doi.org/10.1016/j.molmed.2019.09.007.

46. Florczyk, U., Jazwa, A., Maleszewska, M., Mendel, M., Szade, K., Kozakowska, M., Grochot-Przeczek, A., Viscardi, M., Czauderna, S., Bukowska-Strakova, K., et al. (2014). Nrf2 regulates angiogenesis: effect on endothelial cells, bone marrow-derived proangiogenic cells and hind limb ischemia. Antioxid. Redox Signal. *20*, 1693–1708. https://doi.org/10.1089/ars.2013.5219.

47. Desai, S., Sharma, D., Srinivas, R., Balaji, V., Thakore, V., Bedi, V.S., Jindal, R., Sugumaran, A., Mohanasundaram, S., Gogtay, J., et al. (2024). Mesenchymal stromal cell therapy (REGENACIP((R))), a promising treatment option in chronic limb threatening ischemia - a narrative review. Stem Cell Res. Ther. *15*, 352. https://doi.org/10.1186/s13287-024-03957-0.

48. Taniyama, Y., Morishita, R., Hiraoka, K., Aoki, M., Nakagami, H., Yamasaki, K., Matsumoto, K., Nakamura, T., Kaneda, Y., and Ogihara, T. (2001). Therapeutic angiogenesis induced by human hepatocyte growth factor gene in rat diabetic hind limb ischemia model: molecular mechanisms of delayed angiogenesis in diabetes. Circulation *104*, 2344–2350. https://doi.org/10.1161/hc4401.098470.

49. Zhao, R., Wang, L., Wang, T., Xian, P., Wang, H., and Long, Q. (2022). Inhalation of MSC-EVs is a noninvasive strategy for ameliorating acute lung injury. J. Control Release *345*, 214–230. https://doi.org/10.1016/j.jconrel.2022.03.025.

50. Ning, H., Chen, H., Deng, J., Xiao, C., Xu, M., Shan, L., Yang, C., and Zhang, Z. (2021). Exosomes secreted by FNDC5-BMMSCs protect myocardial infarction by anti-inflammation and macrophage polarization via NF-kappaB signaling pathway and Nrf2/HO-1 axis. Stem Cell Res. Ther. *12*, 519. https://doi.org/10.1186/s13287-021-02591-4.

51. Gao, H., Luo, Z., Jin, Z., Ji, Y., and Ying, W. (2021). Adipose Tissue Macrophages Modulate Obesity-Associated beta Cell Adaptations through Secreted miRNA-Containing Extracellular Vesicles. Cells *10*, 2451. https://doi.org/10.3390/cells10092451.

52. Fedorov, Y., Anderson, E.M., Birmingham, A., Reynolds, A., Karpilow, J., Robinson, K., Leake, D., Marshall, W.S., and Khvorova, A. (2006). Off-target effects by siRNA can induce toxic phenotype. RNA *12*, 1188–1196. https://doi.org/10.1261/rna.28106.

53. Raemdonck, K., Vandenbroucke, R.E., Demeester, J., Sanders, N.N., and De Smedt, S.C. (2008). Maintaining the silence: reflections on long-term RNAi. Drug Discov. Today *13*, 917–931. https://doi.org/10.1016/j.drudis.2008.06.008.

54. Moiani, A., Paleari, Y., Sartori, D., Mezzadra, R., Miccio, A., Cattoglio, C., Cocchiarella, F., Lidonnici, M.R., Ferrari, G., and Mavilio, F. (2012). Lentiviral vector integration in the human genome induces alternative splicing and generates aberrant transcripts. J. Clin. Invest. *122*, 1653–1666. https://doi.org/10.1172/JCI61852.

55. von Kalle, C., Deichmann, A., and Schmidt, M. (2014). Vector integration and tumorigenesis. Hum. Gene Ther. *25*, 475–481. https://doi.org/10.1089/hum.2014.2525.

56. Frangoul, H., Locatelli, F., Sharma, A., Bhatia, M., Mapara, M., Molinari, L., Wall, D., Liem, R.I., Telfer, P., Shah, A.J., et al. (2024). Exagamglogene Autotemcel for Severe Sickle Cell Disease. N. Engl. J. Med. *390*, 1649–1662. https://doi.org/10.1056/NEJMoa2309676.

57. Locatelli, F., Lang, P., Wall, D., Meisel, R., Corbacioglu, S., Li, A.M., de la Fuente, J., Shah, A.J., Carpenter, B., Kwiatkowski, J.L., et al. (2024). Exagamglogene Autotemcel for Transfusion-Dependent beta-Thalassemia. N. Engl. J. Med. *390*, 1663–1676. https://doi.org/10.1056/NEJMoa2309673.

58. Zavitsanou, A.M., Pillai, R., Hao, Y., Wu, W.L., Bartnicki, E., Karakousi, T., Rajalingam, S., Herrera, A., Karatza, A., Rashidfarrokhi, A., et al. (2023). KEAP1 mutation in lung adenocarcinoma promotes immune evasion and immunotherapy resistance. Cell Rep. *42*, 113295. https://doi.org/10.1016/j.celrep.2023.113295.

59. Brumage, L., Best, S., Hippe, D.S., Grunblatt, E., Chanana, P., Wu, F., Lee, M.C., Ying, Z., Ibrahim, A., Chung, J.H., et al. (2025). In vivo functional screens reveal KEAP1 loss as a driver of chemoresistance in small cell lung cancer. Sci. Adv. *11*, eadq7084. https://doi.org/10.1126/sciadv.adq7084.

60. Kim, S.J., Habib, O., Kim, J.S., Han, H.W., Koo, S.K., and Kim, J.H. (2017). A homozygous Keap1-knockout human embryonic stem cell line generated using CRISPR/Cas9 mediates gene targeting. Stem Cell Res. *19*, 52–54. https://doi.org/10.1016/j.scr.2016.12.028.

61. Lee, J.S., Lee, J.Y., Song, D.W., Bae, H.S., Doo, H.M., Yu, H.S., Lee, K.J., Kim, H.K., Hwang, H., Kwak, G., et al. (2020). Targeted PMP22 TATA-box editing by CRISPR/Cas9 reduces demyelinating neuropathy of Charcot-Marie-Tooth disease type 1A in mice. Nucleic Acids Res. *48*, 130–140. https://doi.org/10.1093/nar/gkz1070.

62. Park, J., Lim, K., Kim, J.S., and Bae, S. (2017). Cas-analyzer: an online tool for assessing genome editing results using NGS data. Bioinformatics *33*, 286–288. https://doi.org/10.1093/bioinformatics/btw561.

63. Langmead, B., and Salzberg, S.L. (2012). Fast gapped-read alignment with Bowtie 2. Nat. Methods *9*, 357–359. https://doi.org/10.1038/nmeth.1923.

64. Quinlan, A.R., and Hall, I.M. (2010). BEDTools: a flexible suite of utilities for comparing genomic features. Bioinformatics *26*, 841–842. https://doi.org/10.1093/bioinformatics/btq033.

65. Gentleman, R.C., Carey, V.J., Bates, D.M., Bolstad, B., Dettling, M., Dudoit, S., Ellis, B., Gautier, L., Ge, Y., Gentry, J., et al. (2004). Bioconductor: open software development for computational biology and bioinformatics. Genome Biol. *5*, R80. https://doi.org/10.1186/gb-2004-5-10-r80.

66. Kim, M., Jeon, E.J., Chung, W.G., Kim, H., Kim, E., Lee, S., Lee, J.H., Cho, S.W., and Park, J.U. (2025). 3D Soft Liquid Metal Electrodes for High-Resolution and Long-Term Electromyography in Muscle Trauma Assessment. Adv. Mater. *37*, e2419250. https://doi.org/10.1002/adma.202419250.

67. Jia, J., Jeon, E.J., Li, M., Richards, D.J., Lee, S., Jung, Y., Barrs, R.W., Coyle, R., Li, X., Chou, J.C., et al. (2020). Evolutionarily conserved sequence motif analysis guides development of chemically defined hydrogels for therapeutic vascularization. Sci. Adv. *6*, eaaz5894. https://doi.org/10.1126/sciadv.aaz5894.

68. Kang, B., Shin, J., Park, H.J., Rhyou, C., Kang, D., Lee, S.J., Yoon, Y.S., Cho, S.W., and Lee, H. (2018). High-resolution acoustophoretic 3D cell patterning to construct functional collateral cylindroids for ischemia therapy. Nat. Commun. *9*, 5402. https://doi.org/10.1038/s41467-018-07823-5.

69. Li, M., Luo, X., Lv, X., Liu, V., Zhao, G., Zhang, X., Cao, W., Wang, R., and Wang, W. (2016). In vivo human adipose-derived mesenchymal stem cell tracking after intra-articular delivery in a rat osteoarthritis model. Stem Cell Res. Ther. *7*, 160. https://doi.org/10.1186/s13287-016-0420-2.

YMTHE, Volume *34*

Supplemental Information

Targeted *KEAP1* disruption enhances antioxidant defense and mesenchymal stromal cell therapy for chronic limb-threatening ischemia

Eun Ji Shin, Yuri Choi, Eun Je Jeon, Kang-in Lee, Kyu-jun Lee, Young Ju Son, Min Ji Son, Seokjoong Kim, Seung-Woo Cho, and Jae Young Lee

Table S1. sgRNAs used in this study.

sgRNA	Target sequence - PAM (5' to 3')
sgKEAP1_1	TGCAGTCACAGTGCCCTGAG-GGG
sgKEAP1_2	GAAGGTGCGGTTGCCATGCT-GGG
sgKEAP1_3	TGAAGGTGCGGTTGCCATGC-TGG
sgKEAP1_4	GATCATACCAAGCAGGCCTT-TGG
sgKEAP1_5	TTGGCATCATGAACGAGCTG-CGG
sgKEAP1_6	TGAGGCCAGCACCACCTTGT-GGG
sgKEAP1_7	CATGGCCTTGAAGACAGGGC-TGG
sgKEAP1_8	GTGAACATGGCCTTGAAGAC-AGG
sgKEAP1_9	CCATGTTCACCAACGGGCTG-CGG
sgKEAP1_10	ATGGAGGTGGTGTCCATTGA-GGG
sgKEAP1_11	CCAGGTAGCTGAGCGACTGT-CGG
sgKEAP1_12	GAGGCTTACAACCCCAGTGA-CGG
sgKEAP1_13	TCATGGGGTTGTAACAGTCC-AGG
sgKEAP1_14	ACAACCCCATGACCAATCAG-TGG
sgKEAP1_15	CTGCATCCACCACAACAGTG-TGG
sgAAVS1	ATGGAGCCAGAGAGGATCCT-GGG
sgSHS231	GATGTGCTCACTGAGTCTGA-AGG

Table S2. Representative targeted deep-sequencing reads (5 most frequent indel patterns) from sgKEAP1 and Cas9 treated human MSCs. The sgRNA sequence is underlined, the PAM sequence in blue.

Indel	Local Sequence	Frequency (%)
WT	TGCCGTCGGCGGCTCCCACGG**CTGCATCCACCACAACAGTG****TGG**AGAGGTGAGTGGCAGGGCCTGGGGGT	
-1	TGCCGTCGGCGGCTCCCACGGCTGCATCCACCACA--AGTGTGGAGAGGTGAGTGGCAGGGCCTGGGGGT	57.51
-2	TGCCGTCGGCGGCTCCCACGGCTGCATCCACCACAACA**A**GTGTGGAGAGGTGAGTGGCAGGGCCTGGGGGT	13.27
+1	TGCCGTCGGCGGCTCCCACGGCTGCATCCACC---ACAGTGTGGAGAGGTGAGTGGCAGGGCCTGGGGGT	3.90
-3	TGCCGTCGGCGGCTCCCACGGCTGCATCCAC-----CAGTGTGGAGAGGTGAGTGGCAGGGCCTGGGGGT	3.08
-4	TGCCGTCGGCGGCTCCCACGGCTGCATCCACCACAAC-GTGTGGAGAGGTGAGTGGCAGGGCCTGGGGGT	0.75

Table S3. Potential off-target sites of the sgKEAP1 in the human genome (GRCh38.hg38) revealed by *in silico* analysis.

	Location	Position	Target sequence (5' to 3')	Gene
On-target	Chr19	10491580	CTGCATCCACCACAACAGTGTGG	*KEAP1*
Off1	Chr3	70902810	CTGCATCCACCACcACAGTGAAG	Intergenic
Off2	Chr7	33225786	CTctATCCACCACAACAGTGCAG	BBS9 (Intron)

Table S4. Potential off-target sites of the sgKEAP1 in the human genome (GRCh38.hg38) revealed by *Digenome-seq* analysis.

	Location	Position	Target sequence (5' to 3')	Gene
On-target	chr19	10491580	CTGCATCCACCACAACAGTGTGG	*KEAP1*
Off3	Chr8	135488419	CCCTACTGTGTGGTGGATACAA	KHDRBS3 (Intron)
Off4	Chr7	33225793	CTGCACTGTTGTGGTGGATAGAG	BBS9 (Intron)
Off5	Chr5	18717963	CCCCACTGTTGTTATGATGCAG	Intergenic
Off6	Chr5	128243049	CCCCACTGTTTGGTGGGTGCAG	Intergenic
Off7	Chr4	72924759	CCCCAATCCCCACAACAGTGTGG	Intergenic
Off8	Chr3	117724462	CTTAATCCCCACAACAATGAGG	ENSG00000242816 (Intron)
Off9	Chr14	61334146	CCACCTCAGTGTGTGGTGGAGGCAG	PRKCH (Intron)

Table S5. Sequence of primers used for RTq-PCR.

Gene	Forward (5′-3′)	Reverse (5′-3′)
human *KEAP1*	ATTGGCTGTGTGGAGTTGC	CAGGTTGAAGAACTCCTCTTGC
human *GAPDH*	AGCCACATCGCTCAGACACC	GTACTCAGCGGCCAGCATCG
human *PRDX1*	CTGCCAAGTGATTGGTGCTTCTG	AATGGTGCGCTTCGGGTCTGAT
human *GCLM*	TCTTGCCTCCTGCTGTGTGATG	TTGGAAACTTGCTTCAGAAAGCAG
human *PTGS2*	CGGTGAAACTCTGGCTAGACAG	GCAAACCGTAGATGCTCAGGGA
human *AKR1C3*	CCGAAGCAAGATTGCAGATGGC	GTGAGTTTTCCAAGGCTGGTCG
human *PPIF*	CGACTTCACCAACCACAATGGC	GGTGTTAGGACCAGCATTAGCC
human *BNIP3*	TCAGCATGAGGAACACGAGCGT	GAGGTTGTCAGACGCCTTCCAA
human *FTH1*	TGAAGCTGCAGAACCAACGAGG	GCACACTCCATTGCATTCAGCC
human *TXNRD1*	GTTACTTGGGCATCCCTGGTGA	CGCACTCCAAAGCGACATAGGA
human *NQO1*	CCTGCCATTCTGAAAGGCTGGT	GTGGTGATGGAAAGCACTGCCT
human *SRXN1*	GCAGAGCCTCGTGGACACGAT	ATGGTCTCTCGCTGCAGTTGCT
human *PRNP*	TTCGGCAGTGACTATGAGGACC	TTGTGGTGACCGTGTGCTGCTT
human *SQSTM1*	TGTGTAGCGTCTGCGAGGGAAA	AGTGTCCGTGTTTCACCTTCCG
human *GSR*	TATGTGAGCCGCCTGAATGCCA	CACTGACCTCTATTGTGGGCTTG
human *TXN*	GTAGTTGACTTCTCAGCCACGTG	CTGACAGTCATCCACATCTACTTC
mouse *Gapdh*	TCACCACCATGGAGAAGGC	GCTAAGCAGTTGGTGGTGCA
mouse *Il10*	CGGGAAGACAATAACTGCACCC	CGGTTAGCAGTATGTTGTCCAGC
mouse *Tgfb1*	TGATACGCCTGAGTGGCTGTCT	CACAAGAGCAGTGAGCGCTGAA
mouse *Mrc1*	GTTCACCTGGAGTGATGGTTCTC	AGGACATGCCAGGGTCACCTTT
mouse *Hgf*	GTCCTGAAGGCTCAGACTTGGT	CCAGCCGTAAATACTGCAAGTGG

Table S6. Donor information for human BM-MSCs used for current study

Vendor	CAT#	LOT#	Stock passage	Age(Y)	Sex	Race	>=Positive 90%	5%<=positive
Lonza	PT-2501	19TL155677	P2	31	M	Black	CD29 44 73 90 105 166	CD31 45 DR 19
Lonza	PT-2501	20TL153402	P2	35	M	Caucasian	CD29 44 73 90 105 166	CD14 31 45 DR 19
Lonza	PT-2501	21TL333343	P2	25	M	Black	CD29 73 90 105 166	CD14 31 45 DR 19

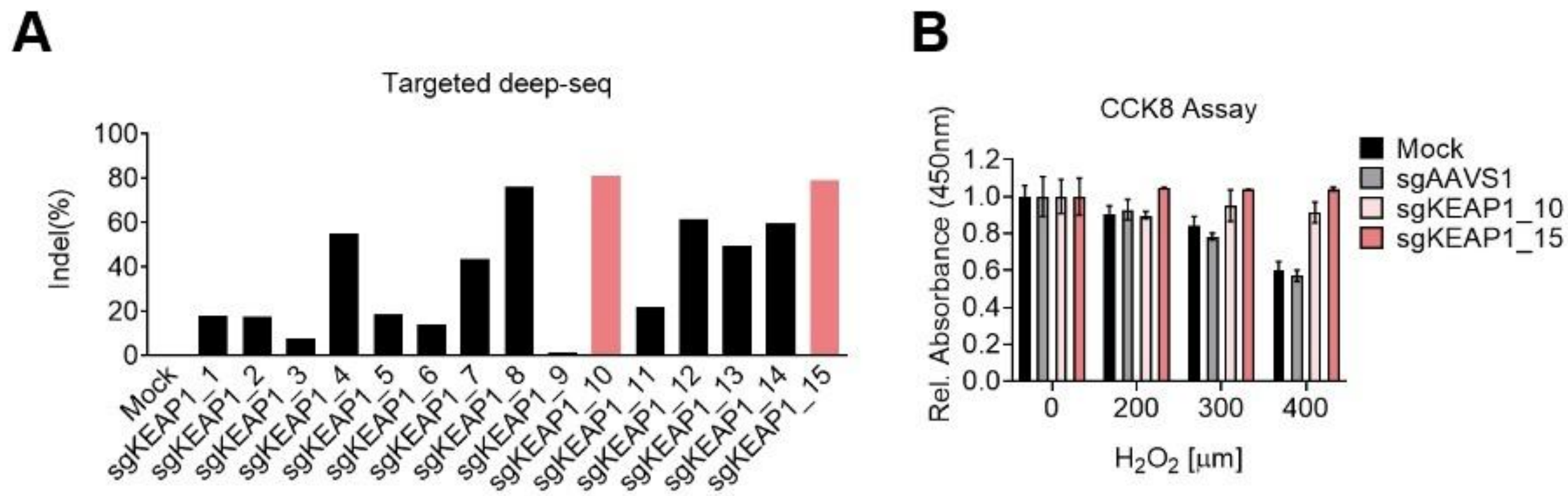

Figure S1. Screening and identification of optimal sgRNA for *KEAP1* gene disruption.

(A) Targeted deep sequencing results showing indel frequencies induced by 15 sgRNAs designed against *KEAP1* in human bone marrow derived MSCs. sgKEAP1_10 and sgKEAP1_15 exhibited the highest editing efficiencies (highlighted in pink) and were selected for downstream validation. **(B)** Cell viability of sgKEAP1_10– and sgKEAP1_15–edited cells evaluated by CCK-8 assay following exposure to increasing concentrations of hydrogen peroxide (n = 2 Mock-transfected and sgAAVS1-edited cells served as controls).

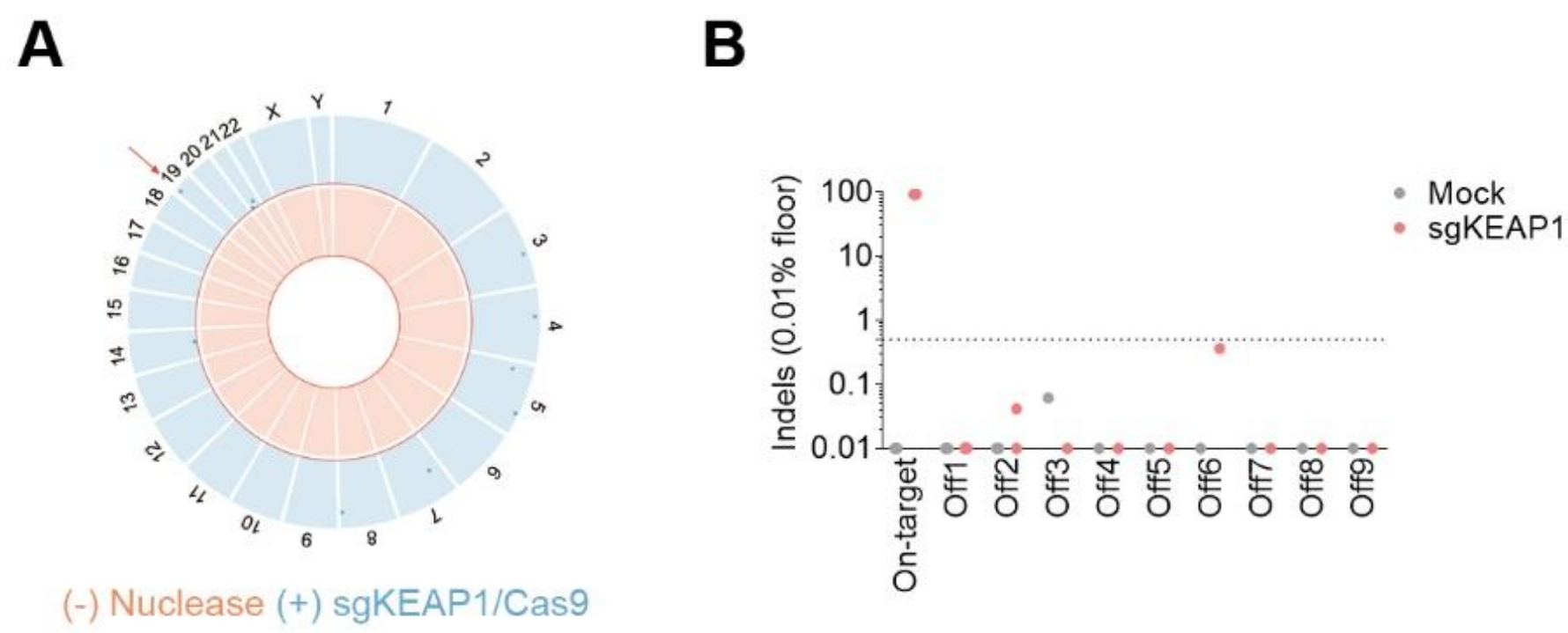

Figure S2. Specificity assessment of *KEAP1* gene editing by sgKEAP1/Cas9.
The overall workflow for off-target evaluation involved genome-wide identification of potential Cas9 cleavage sites by Digenome-seq, followed by targeted deep sequencing to quantitatively validate editing specificity at the on-target and predicted off-target loci. **(A)** Genome-wide off-target profiling by Digenome-seq analysis showing cleavage patterns in the presence (+, blue) or absence (–, orange) of sgKEAP1/Cas9 nuclease. On-target locus on chromosome 19 is indicated in red arrow. **(B)** Targeted deep sequencing of the *KEAP1* on-target site and 9 candidate off-target sites in human BM-MSCs transfected with sgKEAP1 or mock control. Off-target loci were selected based on combined Digenome-seq and *in silico* prediction (≤2 bp mismatch to the sgKEAP1 protospacer). Indel frequencies at all putative off-target sites remained below background threshold (<0.5%), confirming the high specificity of sgKEAP1/Cas9 editing in MSCs.

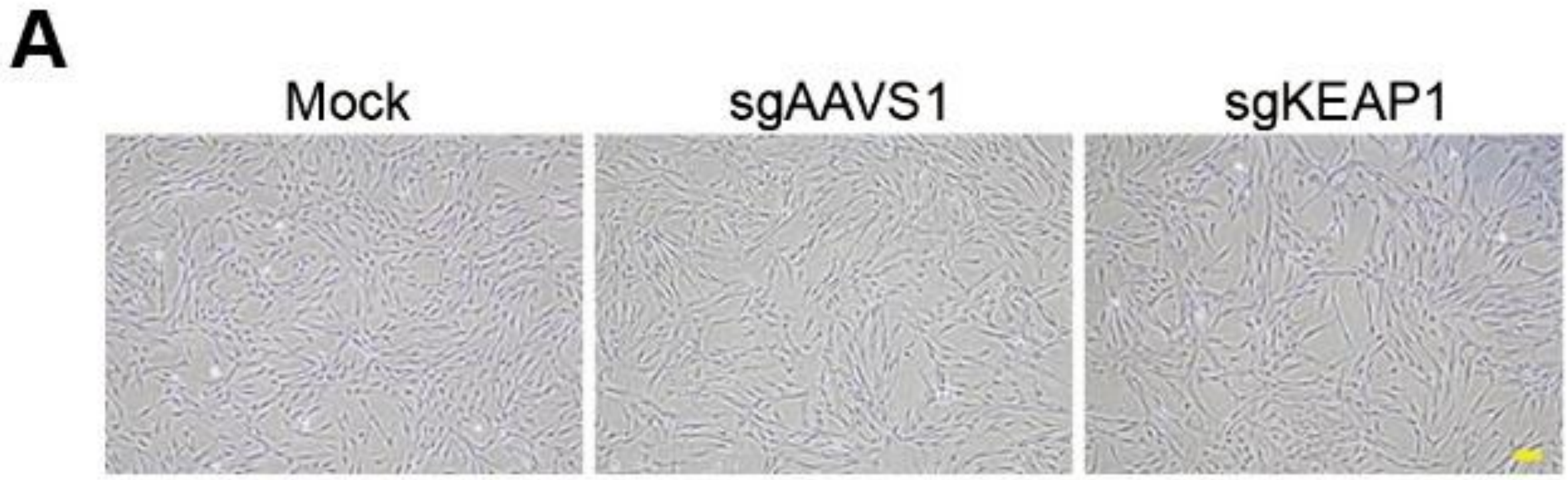

Figure S3. Morphological assessment of MSCs following KEAP1 gene editing.
(A) Representative phase-contrast micrographs of human bone marrow–derived MSCs at day 7 post gene editing. Cells edited with non-targeting control sgAAVS1 or *KEAP1*-specific sgRNA (sgKEAP1) displayed typical fibroblast-like morphology comparable to mock-transfected controls, indicating that *KEAP1* knockout did not alter MSC morphology or gross cellular phenotype. Scale bar, 100 µm.

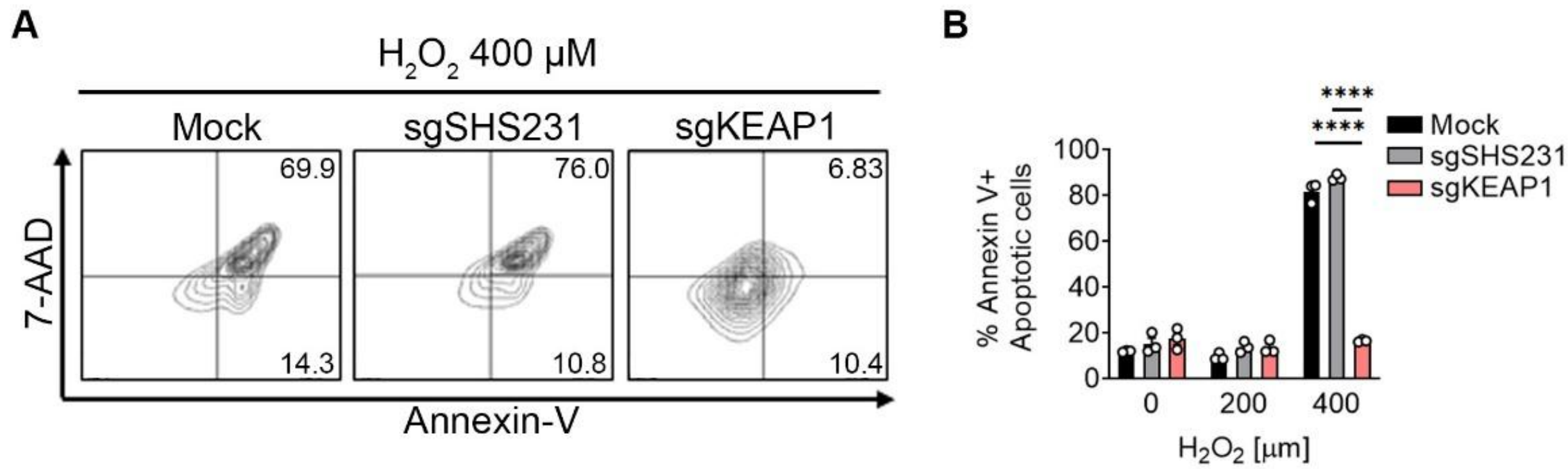

Figure S4. KEAP1 disruption reduces oxidative stress-induced apoptosis in MSCs.
(A) Representative flow cytometric plots of Annexin-V and 7-AAD staining in Mock, sgSHS231, and sgKEAP1 MSCs after 24-hour exposure to 400 µM H_2O_2. (B) Quantification of Annexin-V$^+$ apoptotic cells across increasing H_2O_2 concentrations $n = 3$, ****$P < 0.0001$ by two-way ANOVA with Tukey's post hoc test).

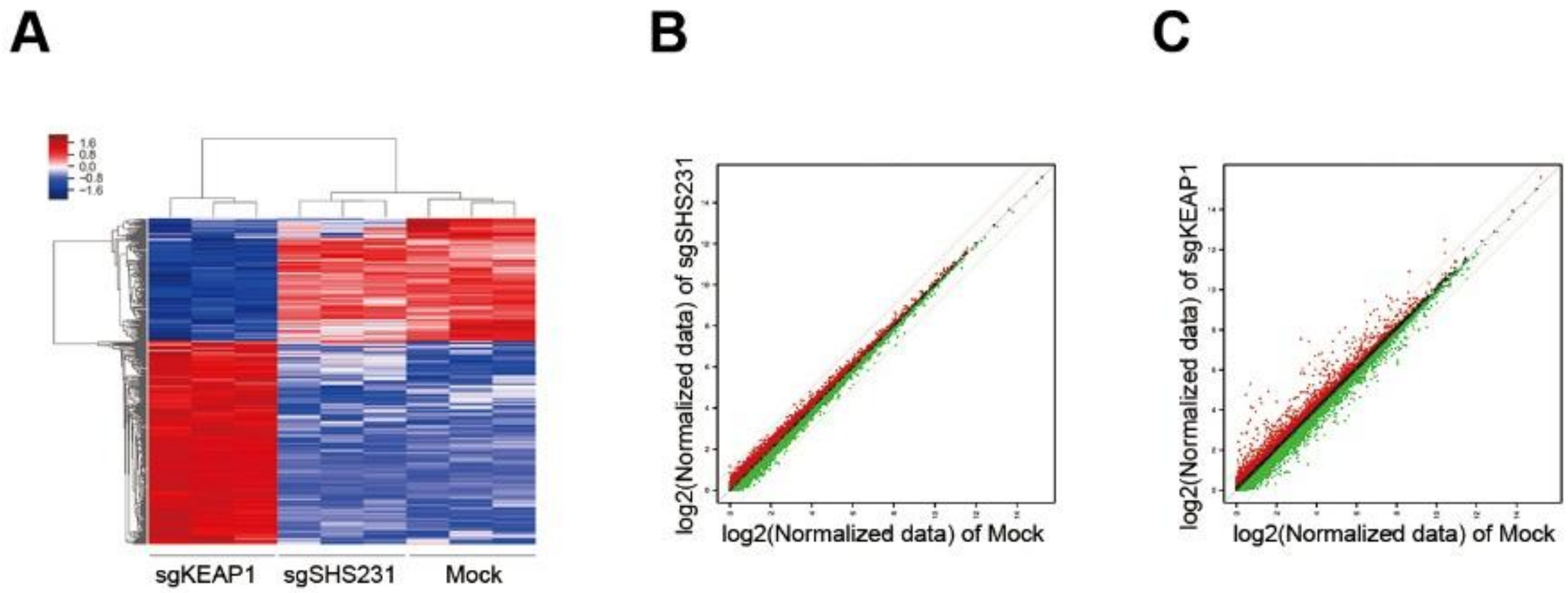

Figure S5. Transcriptomic profiling of KEAP1-edited MSCs by RNA sequencing.
(A) Heatmap displaying hierarchical clustering of differentially expressed genes among *KEAP1*-knockout (sgKEAP1), control (sgSHS231), and mock-transfected MSCs at day 7 post-editing. Each row represents an individual gene, and color scale denotes z-score–normalized expression values (red, upregulated; blue, downregulated). *KEAP1* knockout resulted in a distinct transcriptomic signature characterized by widespread upregulation of antioxidant and cytoprotective genes relative to controls. **(B–C)** Scatter (MA) plots comparing global gene expression profiles of sgSHS231 **(B)** and sgKEAP1 **(C)** versus mock controls. Each dot represents one gene; red and green indicate significantly upregulated and downregulated genes, respectively (fold change >2, adjusted $P<0.05$). *KEAP1* editing induced a broader and more pronounced transcriptomic shift than the non-targeting control, consistent with NRF2 pathway activation and metabolic reprogramming in MSCs.

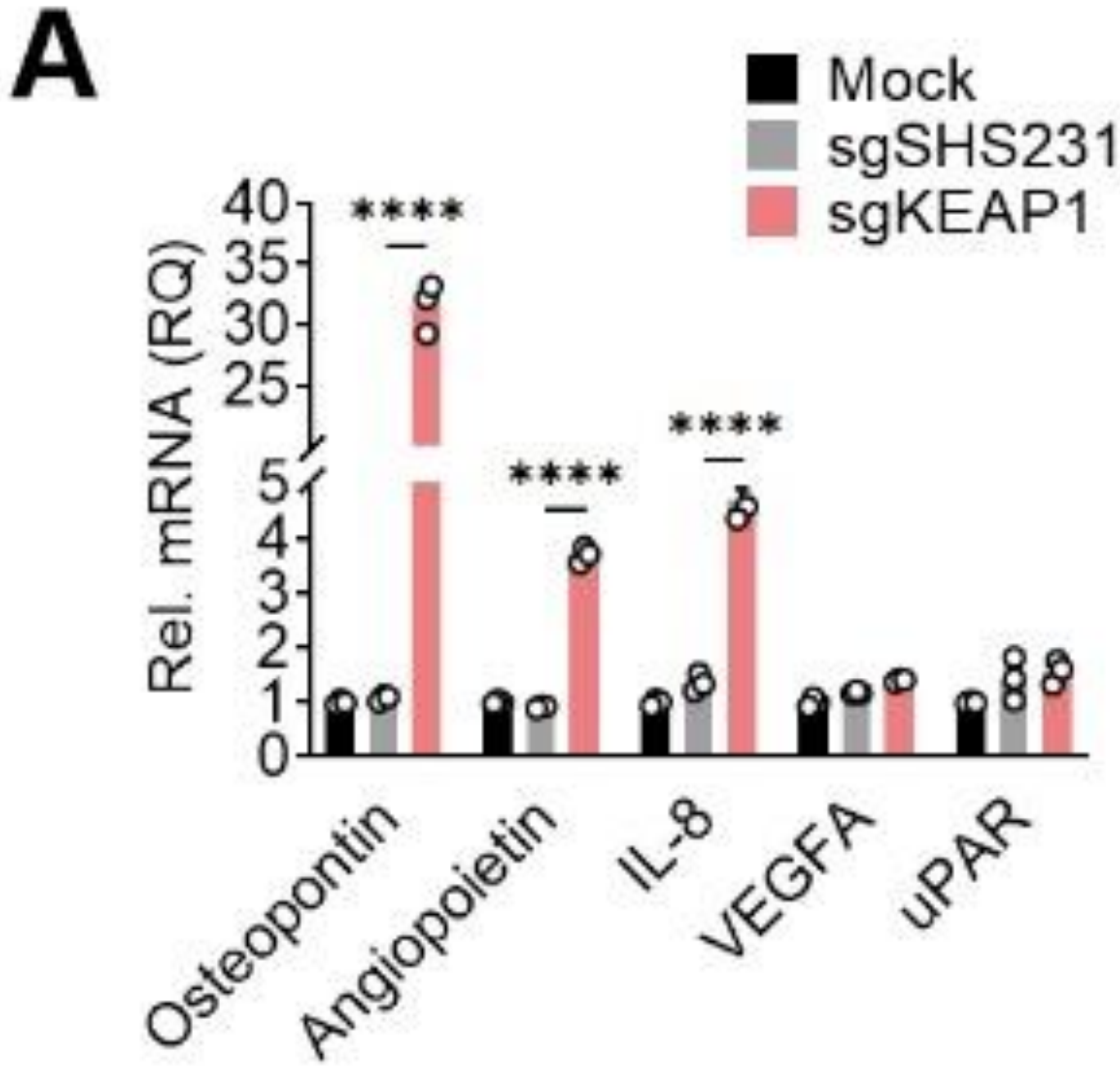

Figure S6. Confirmation of cytokine gene upregulation in sgKEAP1 MSCs.
(A) RT-qPCR analysis of mRNA expression levels corresponding to cytokines identified in Fig. 4 (n = 3 biological replicates; one-way ANOVA with Tukey's post hoc test; ***$P < 0.0001$).

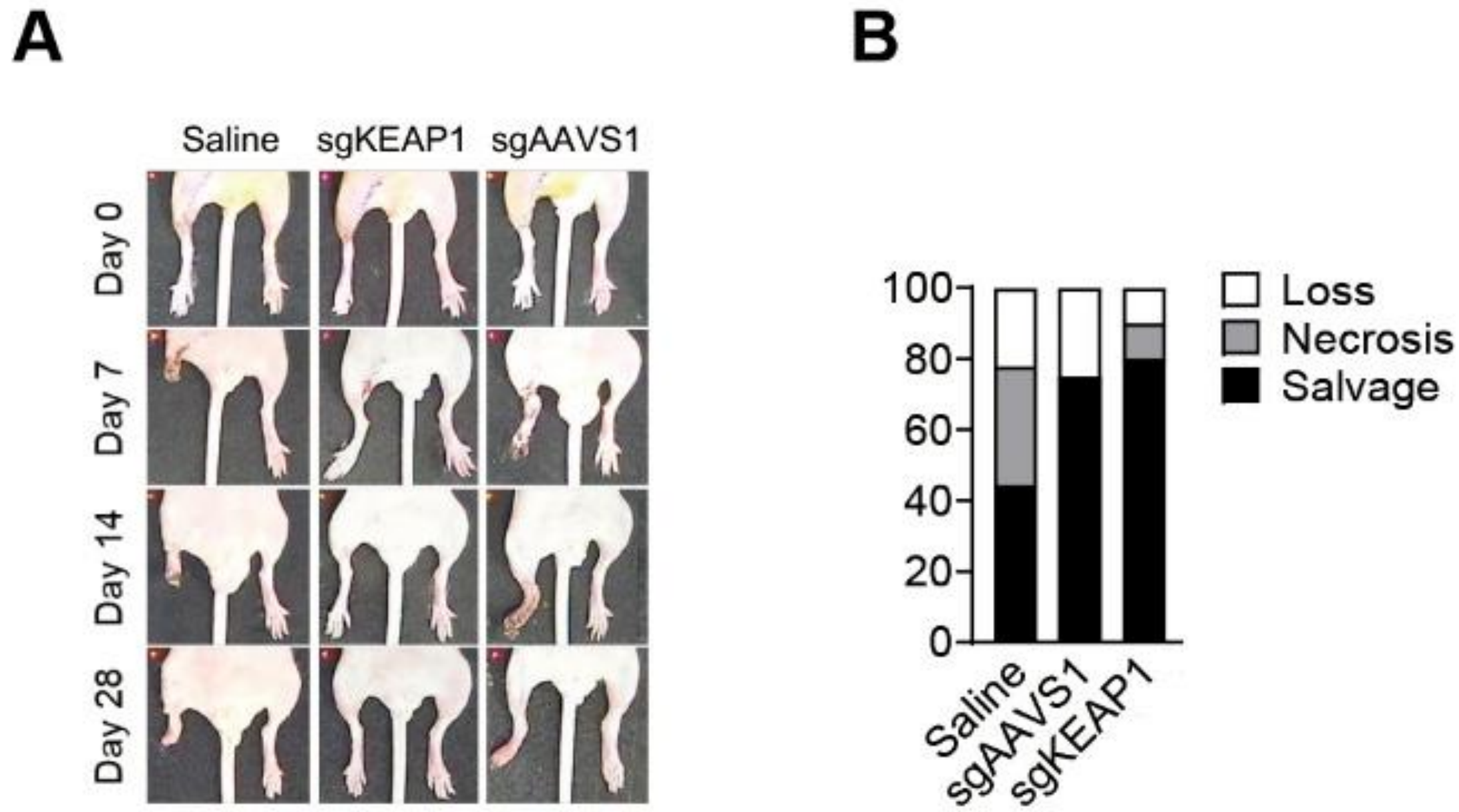

Figure S7. KEAP1-edited MSCs attenuate ischemic tissue necrosis *in vivo*.
(A) Representative macroscopic images of hindlimbs from each treatment group at indicated time points (days 0–28). **(B)** Quantification of limb salvage outcomes based on necrosis scoring at day 28. sgKEAP1-treated limbs exhibited a higher rate of tissue salvage and reduced necrosis compared with sgAAVS1 and saline controls (*n* = 8-10 mice per group).
